# Supplementary material for: Barriers to equitable access to quality trauma care in Rwanda: a qualitative study
Source: BMJ Open. 2023 Sep 28;13(9):e075117. doi: 10.1136/bmjopen-2023-075117 (PMC10546151; doi:10.1136/bmjopen-2023-075117)
Supplement: Supplementary data [file bmjopen-2023-075117supp001.pdf]

Supplementary Information

Discussion Guides

1. Service Users Focus Group Discussion Guide

First, I would like to thank you for attending this discussion.

The recordings will be anonymous, and we don't want you to say your name at the start, but we have given each of you a pseudonym (made up name), and will go around the table in turn asking you to say your pseudonym, what age you are, the area in which you live, This will help us to identify individual voices when we come to write up the interviews.

This group discussion is to understand what would happen in your community if someone suffered an injury.

We would like to think particularly about four different aspects of what would happen, these aspects are seeking care (the time from an injury happens to taking action to get care), reaching care (the time until a facility or hospital that can treat the injury is reached), receiving quality care (getting good quality treatment for the injury at a facility or hospital), and remaining in care (for example, attending follow up at out-patients or going to rehabilitation).

Seeking care

Firstly, we wish to know what do people do straight after injuries occur?
Does the severity of the injury (how bad it is) determine what people do?

For people who need to go and get treatment, what happens after injuries?
What factors do you think make people more likely to seek health care?
What factors do you think make people less likely to seek health care? Prompts, if necessary For example:

- Knowledge of availability of healthcare?
- Trust in healthcare?

- Fear of costs?
- Availability of a phone?

If people don't seek healthcare what do they do instead?

Reaching Care

Now we wish to know what a person does after an injury has occurred and it has been decided to seek health care.
Does what a person does depend on the severity of the injury?

What factors would make it more likely to reach a place of care quickly?
What factors make it less likely to reach a place of care quickly? Prompts, if necessary For example:

- Ambulance availability?
- Road conditions?
- Personal safety?
- Costs?

Receiving care

How about the care received after the injured person has made it to a place of care? Would you consider that this will be good quality care? Prompts, if necessary For example:

- Will the injured person get the correct treatment?
- Will they be treated with respect?
- Will they have to wait for a long time?

What factors do you think make it difficult for injured people to receive good quality care? What factors make it more likely they will receive good quality care? Prompts, if necessary For example:

- Qualifications of staff at the facility?
- Availability of equipment at the facility?

- Personal contacts with staff at the facility?
- Costs of care?

Remaining in care

This is the final stage in the care-seeking process and we would like you to tell us about what happens for people who are discharged from hospital or, when their injury has been mostly treated, but, they have been asked to come back for more care, like out-patient appointments or physiotherapy (rehabilitation)

Do most people who have been asked to return to (or remain in) care actually do so?
What are the barriers to people remaining in care?
What are the facilitators to people remaining in care?

Prompts, if necessary

For example:

- Distances needed to travel?
- Other family commitments?

Solutions

Finally, what do you think could be done to improve access to quality care for people who are injured in your area?

Does anyone have any questions for us?
Thank you

2. **Service User In-Depth Interview Topic Guide**

First, I would like to thank you for attending this interview.

The recordings will be anonymous, and we don't want you to say your name at the start, but we have given you a pseudonym (made up name) that you will use during this interview.

This interview is to understand what would happen in your community if someone suffered an injury.

I would like to think particularly about four different aspects of what would happen, these aspects are seeking care (the time from an injury happens to taking action to get care), reaching care (the time until a facility or hospital that can treat the injury is reached), receiving quality care (getting good quality treatment for the injury at a facility or hospital), and remaining in care (for example, attending follow up at out-patients or going to rehabilitation).

I wish to know about your experiences and also your thoughts on whether others in your community have similar experiences.

I realize that you have had an injury in the last 6 months, could you tell us about what happened to cause the injury?

Where were you when the injury happened?

How about after your injury happened, what did you do?
Prompts, if needed:

- Did you think that you needed help?
- Was anyone there to help?
- Were you able to telephone for help?

Did you think that you needed to go and get treatment?

(If not, why not?)

Were there any things that stopped you trying to get treatment?

Prompts, if needed

- Knowledge of availability of healthcare?
- Trust in healthcare?
- Fear of costs?
- Availability of a phone?
- No time to go to healthcare?

Where did you first go to for treatment?

Prompts, if needed:

- Is that a clinic or a hospital?
- How far away was it?
- How long did it take to get there?
- If it took a long time to reach the care, why was that?

Was that the only place you went to, or did you need to (or were you referred to) another place to get care?

Note to interviewer: please explore how many places the person needed to go to in order to get definitive care

Were there any things in particular that delayed you getting to a hospital or clinic for care?

Prompts, if needed:

- Ambulance availability?
- Road conditions?
- Personal safety?
- Costs?

How about the care that you got in the facilities that you attended – do you think that the medical treatment was good?

Prompts, if needed:

- Were you seen quickly?
- Did the doctors and nurses seem competent?
- Do you think that they had all that they needed to treat your injury well?

How about how they treated you?

Prompts, if needed:

- Were you treated with respect?
- Did the staff explain things to you?

How about costs of care?

Prompts, if needed:

- Did you have to pay for care?
- Did you have to borrow or sell anything to pay for care?

Were there any things in particular that stopped you getting the right treatment in the hospital?

Prompts if needed:

- Family commitments meant had to shorten stay?
- Costs of care?
- Too far for relatives to travel to visit?

After your injury was treated, were you asked to go back to see healthcare workers about your injury? For example, doctors in a clinical appointment, or physiotherapists for rehabilitation?

If so, how far did you have to travel to get to these appointments?

Did you manage to keep all of the appointments?

Were there any things in particular that you felt limited your ability to keep all these appointments? Prompts, if needed:

- Travel time?
- Costs?
- Other commitments?
- Thought there was no need?

Are you now back to your full fitness, do you think?

Do you think anything could have been done better?

Now that's the end of this discussion, thank-you for telling us about your experiences. Now, we would be grateful if we could ask you some other questions about your time in the hospital or clinic. It will take another ten minutes.

Note to interviewer ask I-PAHC and/or O-PAHC questionnaire depending on whether the participant was admitted to hospital or was seen in a clinic, or both.

Do you have any questions for me?

Thank you

3. **Community Leaders Focus Group Discussion Guide.**

First, I would like to thank you for attending this discussion.

The recordings will be anonymous, and we don't want you to say your name at the start, but we have given each of you a pseudonym (made up name), and will go around the table in turn asking you to say your pseudonym, what age you are, the area in which you live, and what your job is. This will help us to identify individual voices when we come to write up the interviews.

This group discussion is to understand what would happen in your community if someone suffered an injury.

Please based what you say on what you have observed in your community or experienced yourself.

We would like to think particularly about four different aspects of what would happen, these aspects are seeking care (the time from an injury happens to taking action to get care), reaching care (the time until a facility or hospital that can treat the injury is reached), receiving quality care (getting good quality treatment for the injury at a facility or hospital), and remaining in care (for example, attending follow up at out-patients or going to rehabilitation).

To help you think about this subject, we have provided the following examples:

1) A 19 year old girl who slipped off the roof at home hitting her head on the ground and then became very drowsy.
2) A man who has severe pain after being stabbed in the stomach with a knife after an argument in the street.
3) A farmer who was kicked him in the chest by his cow and has difficulty in breathing.
4) A motorcyclist who was hit by another vehicle has a wound and leg swelling with severe pain.

Seeking care

Firstly, we wish to know what would people do straight after injuries occur?
Does the severity of the injury (how bad it is) determine what people do?

For people who need to go and get treatment, what happens after injuries?
What factors do you think make people more likely to seek health care?
What factors do you think make people less likely to seek health care? Prompts, if necessary For example:

- Knowledge of availability of healthcare?
- Trust in healthcare?
- Fear of costs?
- Availability of a phone?
- Preference for traditional or community healers?

If people don't seek healthcare what do they do instead?

Reaching Care

Now we wish to know what a person does after an injury has occurred and it has been decided to seek health care.
Does what a person does depend on the severity of the injury?

What factors would make it more likely to reach a place of care quickly?
What factors make it less likely to reach a place of care quickly? Prompts, if necessary For example:

- Ambulance availability?
- Road conditions?
- Personal safety?
- Costs?

Receiving care

How about the care received after the injured person has made it to a place of care? Would you consider that this will be good quality care? Prompts, if necessary For example:

- Will the injured person get the correct treatment?
- Will they be treated with respect?
- Will they have to wait for a long time?

What factors do you think make it difficult for injured people to receive good quality care? What factors make it more likely they will receive good quality care? <u>Prompts, if necessary</u> For example:

- Qualifications of staff at the facility?
- Availability of equipment at the facility?
- Personal contacts with staff at the facility?
- Costs of care?

Remaining in care

This is the final stage in the care-seeking process and we would like you to tell us about what happens for people who are discharged from hospital or, when their injury has been mostly treated, but, they have been asked to come back for more care, like out-patient appointments or physiotherapy (rehabilitation)

Do most people who have been asked to return to (or remain in) care actually do so?
What are the barriers to people remaining in care?
What are the facilitators to people remaining in care?

<u>Prompts, if necessary</u>
For example:

- Distances needed to travel?
- Other family commitments?

Solutions

Finally, what do you think could be done to improve access to quality care for people who are injured in your area?

Does anyone have any questions for us?
Thank you
