# Supplementary material for: Barriers to equitable access to quality trauma care in Rwanda: a qualitative study
Source: BMJ Open. 2023 Sep 28;13(9):e075117. doi: 10.1136/bmjopen-2023-075117 (PMC10546151; doi:10.1136/bmjopen-2023-075117)
Supplement: Author's manuscript [file bmjopen-2023-075117.draft_revisions.pdf]

BMJ Open is committed to open peer review. As part of this commitment we make the peer review history of every article we publish publicly available.

When an article is published we post the peer reviewers' comments and the authors' responses online. We also post the versions of the paper that were used during peer review. These are the versions that the peer review comments apply to.

The versions of the paper that follow are the versions that were submitted during the peer review process. They are not the versions of record or the final published versions. They should not be cited or distributed as the published version of this manuscript.

BMJ Open is an open access journal and the full, final, typeset and author-corrected version of record of the manuscript is available on our site with no access controls, subscription charges or pay-per-view fees (http://bmjopen.bmj.com).

If you have any questions on BMJ Open's open peer review process please email info.bmjopen@bmj.com

BMJ Open

Equitable access to quality trauma systems in Lower and Middle-Income Countries: A qualitative study assessing gaps and developing priorities in Rwanda

Journal:	*BMJ Open*
Manuscript ID	bmjopen-2023-075117
Article Type:	Original research
Date Submitted by the Author:	26-Apr-2023

Complete List of Authors:	Nzasabimana, Pascal; University of Rwanda, Single Project Implementation Unit<br>Ignatowicz, Agnieszka ; University of Birmingham, Institute of Applied Health Research<br>Alayande, Barnabas; University of Global Health Equity, Center for Equity in Global Surgery<br>Abdul-Latif , Abdul-Malik ; University of Birmingham, Institute of Applied Health Research<br>Odland, Maria Lisa; University of Birmingham, Institute of Applied Health Research; St Olav's Hospital, Trondheim University Hospital, Department of Obstetrics and Gynecology<br>Davies, Justine ; University of Birmingham, Institute of Applied Health Research; Stellenbosch University, Department of Global Health, Centre for Global Surgery<br>Bekele, Abebe; University of Global Health Equity, Center for Equity in Global Surgery<br>BYIRINGIRO, JEAN CLAUDE; University of Rwanda College of Medicine and Health Sciences, School of Medicine and Pharmacy

Keywords:	Health Services Accessibility, Orthopaedic & trauma surgery < SURGERY, Organisation of health services < HEALTH SERVICES ADMINISTRATION & MANAGEMENT

SCHOLARONE™
Manuscripts

BMJ

I, the Submitting Author has the right to grant and does grant on behalf of all authors of the Work (as defined in the below author licence), an exclusive licence and/or a non-exclusive licence for contributions from authors who are: i) UK Crown employees; ii) where BMJ has agreed a CC-BY licence shall apply, and/or iii) in accordance with the terms applicable for US Federal Government officers or employees acting as part of their official duties; on a worldwide, perpetual, irrevocable, royalty-free basis to BMJ Publishing Group Ltd ("BMJ") its licensees and where the relevant Journal is co-owned by BMJ to the co-owners of the Journal, to publish the Work in this journal and any other BMJ products and to exploit all rights, as set out in our licence.

The Submitting Author accepts and understands that any supply made under these terms is made by BMJ to the Submitting Author unless you are acting as an employee on behalf of your employer or a postgraduate student of an affiliated institution which is paying any applicable article publishing charge ("APC") for Open Access articles. Where the Submitting Author wishes to make the Work available on an Open Access basis (and intends to pay the relevant APC), the terms of reuse of such Open Access shall be governed by a Creative Commons licence – details of these licences and which Creative Commons licence will apply to this Work are set out in our licence referred to above.

Other than as permitted in any relevant BMJ Author's Self Archiving Policies, I confirm this Work has not been accepted for publication elsewhere, is not being considered for publication elsewhere and does not duplicate material already published. I confirm all authors consent to publication of this Work and authorise the granting of this licence.

For peer review only

Equitable access to quality trauma systems in Lower and Middle-Income Countries: A qualitative study assessing gaps and developing priorities in Rwanda

Pascal Nzasabimana[1], Agnieszka Ignatowicz[2], Barnabas Alayande[3,4], Abdul-Malik Abdul-Latif[2], Maria Lisa Odland[2,8,9,10], Justine I Davies[2,5,6*], Abebe Bekele[3*], Jean Claude Byiringiro[7*].

1. Single Project Implementation Unit, University of Rwanda, Kigali, Rwanda
2. Institute of Applied Health Research, University of Birmingham, Birmingham, UK
3. Center for Equity in Global Surgery, University of Global Health Equity, Kigali, Rwanda
4. Program in Global Surgery and Social Change, Harvard Medical School, Boston, USA
5. Centre for Global Surgery, Department of Global Health, Stellenbosch University, Cape Town, South Africa
6. Medical Research Council/Wits University Rural Public Health and Health Transitions Research Unit, Faculty of Health Sciences, School of Public Health, University of the Witwatersrand, Johannesburg, South Africa
7. School of Medicine and Pharmacy, University of Rwanda, Kigali, Rwanda
8. Department of Obstetrics and Gynecology, St Olav's Hospital, Trondheim University Hospital, Trondheim, Norway
9. Malawi-Liverpool-Wellcome Trust Research Institute, Blantyre, Malawi
10. Institute of Life Course and Medical Sciences, University of Liverpool, Liverpool, United Kingdom

For peer review only

Correspondence to Dr Agnieszka Ignatowicz, Institute of Applied Health Research, University of Birmingham, Birmingham, B15 2TT, United Kingdom. Email: a.m.ignatowicz@bham.ac.uk

*Co-senior authors

For peer review only

Abstract

Objectives: Using the "Four Delay" framework, our study aimed to identify and explore barriers to accessing quality injury care from the injured patients', caregivers', and community leaders' perspectives.

Design: A qualitative study assessing barriers to trauma care comprising twenty in-depth semi-structured interviews and four focus group discussions were conducted. The data were analyzed thematically.

Setting: This qualitative study was conducted in Rwanda's rural Burera District, located in the Northern Province, and in Kigali City, the country's urban capital, to capture both the rural and urban population's experiences of being injured.

Participants: Purposively selected participants were individuals from urban and rural communities who had accessed injury care in the previous six months or cared for the injured people, and community leaders. Fifty-one participants, 13 females and 38 males ranging from 21 to 68 years of age participated in interviews and focus group discussions. Thirty-six (71%) were former trauma patients with a wide range of injuries including fractured long bones (9, 45%), other fractures, head injury, polytrauma (3, 15% each), abdominal trauma (1, 5%), and lacerations (1, 5%), while the rest were caregivers and community leaders.

Results: Multiple barriers were identified cutting across all levels of the "Four Delays" framework, including barriers to seeking, reaching, receiving, and remaining in care. Key barriers mentioned by participants in both interviews and focus group discussions were: lack of community health insurance, limited access to ambulances, insufficient number of trauma care specialists, and a high volume of trauma patients. The rigid referral process and lack of

For peer review only

decentralized rehabilitation services were also identified as significant barriers to accessing quality care for injured patients.

Conclusions: Future interventions to improve access to injury care in Rwanda must be informed by the identified barriers along the spectrum of care, from the point of injury to receipt of care and rehabilitation.

Keywords: Health Services Accessibility, Wounds and injuries, Rwanda

Strengths and limitations of this study:

- This study uses a more comprehensive “Four Delay” framework (an extension of the “Three Delay” framework) to assess trauma care access, comprising delays in seeking, reaching, receiving, and remaining in care.
- A multi-stakeholder perspective of delay is obtained by triangulating the service user, caregiver, and community leader’s perspectives.
- Wide range of participants from both urban and rural communities of Rwanda were interviewed using both individual in-depth interviews and focus group discussions conducted in the local language.
- To ensure rigor, all transcripts of interviews and focus group discussions were coded in parallel by a context and local language fluent member and qualitative data experts.
- As a limitation to generalizability, we recognize that barriers may differ slightly in other urban and district settings, and that most (75%) participants were male. Whilst this may mean that we missed barriers mostly experienced by females, it should be noted that the majority of injured patients in Rwanda are male.

Funding Statement

For peer review only

Funding for this study was provided by the UK National Institute of Health Research (NIHR), award number 130036. The views expressed in this publication are those of the author(s) and not necessarily those of the NIHR or the UK government.

Competing interests statement

None declared.

INTRODUCTION

Historically, global health initiatives have prioritized infectious diseases. However, in 2019 alone, the world recorded 4.3 million deaths from trauma, more deaths than malaria, tuberculosis, and AIDS combined (1). This burden is heaviest in Low- and Middle- Income Countries (LMICs), where injury rates are high, trauma systems are underdeveloped, and patients requiring emergency care are young and economically active (2). Up to 11% of deaths in Rwanda and 22% of deaths in the largest urban city of Kigali, housing 1,135,428 people according to the 2012 population census, are due to injury, with the most common cause being road traffic crashes (3)(4). Although injury prevention initiatives are necessary, identifying health system deficiencies and barriers that hinder access to safe, affordable, timely trauma care is key to reducing preventable deaths from people who are inevitably injured. A South African study suggests that strengthening health systems by focusing on inter-facility transfer systems would reduce up to 59% of preventable trauma deaths resulting from delays in receiving care. In addition, the same study argues that 23% and 18% of deaths from injury can be prevented by mitigating delays in seeking and reaching trauma care, respectively (5). Identifying and characterizing specific barriers to care is crucial for developing and implementing successful interventions, and reducing the burden of injury (6).

For peer review only

The "Three Delay" framework was developed originally to identify and mitigate barriers to maternal and child care but has been successfully applied to emergency trauma care and injury care (7). A systematic review and evidence synthesis mapping this framework to injury health system assessments found that approximately 79% of studies focused on delays within facilities, 46% assessed delays in reaching care, and only 10% assessed delays in seeking care (8). Research from sub-Saharan Africa comprised 40.1% of the studies assessed in this systematic review. A more comprehensive "Four Delay" framework for assessing trauma care access has been suggested, comprising delays in seeking, reaching, receiving, and remaining in care (9). This more comprehensive model emphasizes the need for holistic care, post-discharge physiotherapy, and other rehabilitation for the injured patient. In a workshop carried out to map barriers to trauma care in Rwanda using the four-delay framework, health providers, academia and government representatives identified 19 high-priority barriers faced by trauma patients and prioritized the top four. Three of the prioritized four were related to receiving care, but most of the barriers cut across multiple components of the four-delay model (9). However, the perspectives of the injured patients have not yet been explored. Our study focused on identifying and exploring barriers to accessing quality care from the perspective of the injured patients to develop priorities for trauma system interventions in Rwanda.

For peer review only

METHODS

Patient and Public Involvement Statement

This study was preceded by a National Trauma Symposium in 2019 organized by the Rwanda Ministry of Health and partners, where over 100 community stakeholders including representatives of the health ministry, Rwanda National Police, local non-governmental organizations, universities, emergency respondents, students, and medical practitioners identified access to trauma care as a national research priority (10). This work was also

premised on a two-day workshop held in Kigali, Rwanda in May 2019 with representation from community, academia, and government which aimed to map barriers to accessing care from injury to rehabilitation and is in line with public priorities (11,12). Injured patients, caregivers and other stakeholders were involved in the design and execution of the study.

Researcher characteristics and reflexivity

Four of the eight authors live and work in Rwanda and served as context experts. Of these four, three are surgical providers who provide various degrees of trauma care and leadership to the trauma system. Overall leadership for the work in Rwanda was provided by a Rwandan trauma surgeon. Interviews were conducted by an investigator who is from rural Rwanda, speaks Kinyarwanda, and has lived experience of the health system and delays. There was adequate gender balance on the research team. Two UK-based investigators are qualitative method experts. The team is experienced in such studies as seven of the eight investigators were involved in similar surgical systems assessments in Ghana and South Africa.

Design

Using a grounded theory qualitative approach and a constructivist research paradigm, this qualitative study was conducted in Rwanda's rural Burera District, located in the Northern Province, and in Kigali City, the country's urban capital, to capture both the rural and urban population's experiences of being injured. Participants were chosen on purpose from urban and rural communities who had received injury care in the previous six months or cared for injured people, as well as community leaders. Twenty in-depth semi-structured interviews and four focus group discussions were conducted. The data was analyzed thematically.

Data source

For peer review only

A list of patients treated for traumatic injuries in the last six months was obtained using information obtained from hospital admission registers at the emergency department and surgical wards of Centre Hospitalier Universitaire de Kigali (CHUK), Rwanda's leading center for tertiary trauma care provision, and Butaro District Hospital, a district facility in northern Rwanda. A list of community leaders within the facilities' catchment area was also obtained through the administration departments of both hospitals. Participants for the twenty in-depth interviews and two focus group discussions were drawn from the hospital register if they were over the age of 18 and had either received injury care themselves within the previous six months or had cared for trauma victims who had received care. Local religious leaders, business leaders, service provider leadership, traditional healers, and local government authority figures participated in two community leaders focus group discussions. Individuals under the age of 18 or with intellectual impairments that would limit full informed consent and participation, as well as service users assessing care during the study period, were excluded. Equally rural and urban distributed, individual interviews lasted 30 minutes on average, and six to nine people's focus groups discussions lasted between one and one and a half hours.

Data analysis

Audio recordings of interviews and focus group discussions were made, transcribed, and translated into English before being uploaded to QSR International's NVivo 12 qualitative data analysis software for analysis (13). Thematic analysis of data was performed, as well as inductive and deductive coding. Themes were developed iteratively with reference to the Four Delay framework and according to whether they represent the delay from seeking, reaching, receiving, or remaining in care (9). We defined the first delay (delay in seeking care) as delays from the time the injury occurred to the time a decision was made to seek medical care. The second delay (delay in reaching care) referred to delays from the time a decision was made to

For peer review only

seek medical care to the time when an appropriate medical facility capable of managing the particular injury (prior to registration) was accessed. The third delay (delay in receiving care) was defined as delays beginning from the point of entering a capable facility to the point of receiving definitive management of the injury. The fourth delay (delays in remaining in care) was defined as delays from the point of having received definitive care to the point of final discharge from follow-up. Remaining in care encompassed the time periods of continued in-hospital care beyond definitive care, discharge, and physical therapy (9). To ensure rigor, all transcripts of interviews and focus group discussions were coded in parallel by a context and local language fluent member (PN) and qualitative data experts (AI and MLO). The analysis team met on a regular basis to discuss the coding process. Any conflicts that arose during the independent coding process were resolved by group consensus. The analysis procedure was the same for interviews and focus groups. Following coding and the identification of initial categories, data from interviews and focus groups were combined. The final list of themes was reviewed and agreed upon by the entire investigator team.

For peer review only

RESULTS

A total of 51 participants, 28 from the rural Burera District and 23 from the urban Kigali city, participated in the interviews and focus group discussions. Of all the participants, 13 were female and 38 were male, ranging from 21 to 68 years of age. Thirty-six (71%) of the participants were patients (service users), while the rest were caregivers and community leaders. For the individual in-depth interviews, 10 patients (50%) were from urban Kigali, and 10 (50%) from rural Burera. They presented with a wide range of injuries including fractured long bones (9, 45%), other fractures (3, 15%), head injury (3, 15%), polytrauma (3, 15%), abdominal trauma (1, 5%), and lacerations (1, 5%). There was no statistically significant difference in the patient's type of injury by rural or urban location (X^2 =8.444, df =5, p =0.133).

Sixty barriers were identified across all four delays. The most mentioned barriers were the insufficient number of competent trauma care providers, the rigid referral process, and the facility-related delays in delivery of care. Table 1 shows the barriers to injury care identified by participants across all delays.

Table 1: Barriers to injury care identified by participants across all delays

**Delay**		
	Categories of barriers	
		List of barriers
**First delay (delay in seeking care)**		
	Community health workforce related delays	
		Perceived sufficiency of respondent's interventions
	Community leadership related delays	
		Community conflict management process
	Health financing related delays	
		Pre-injury financial challenges
		Limited comprehensiveness of the insurance system
	Health services delivery related delays	
		Perceived inaccessibility of the "right" hospital or acute care facility
		Lack of awareness of healthcare and injury care systems
		Lack of confidence in healthcare system
		Preference for alternative healthcare
	Knowledge on health information related delays	
		Underestimation of the severity of the injury
		Religious beliefs
		Respondent rumors about seeking injury care
		Shared decision-making (contribution of community health workers and village leaders)
**Second delay (delay in reaching care)**		
	Health care services access related delays	
		Limited geographical coverage of ambulance services

For peer review only

		Cost of transport to hospital and between hospitals
		Unavailability of transportation
		Limited access to ambulances
		Ignorance of ambulance call process and emergency numbers
		High urban road traffic density
		Time of the day of injury
	Infrastructure related delays	
		Poor road infrastructure
	Health financing related delays	
		Fear of spending on private transport cost
		Absence of personal health insurance
	Community leadership related delays	
		Unwillingness of passers-by to assist
		Fear of legal complications for intervening private car owners
		Community organization required for reaching facilities
		Lack of social assistance and support
		Accident investigation process by the police
	Community health workforce related delays	
		Delays in arrival of emergency respondents
		Occurrence of multiple road traffic accidents
		Poorly coordinated inter-facility transfers
**Third delay (delay in receiving care)**		
	Health financing related delays	
		Lack of financial means
		Absence of health insurance
		Cumbersome facility invoicing and cost recovery system
		Unavailability of some community health insurance staff during work hours
		Unaffordable costs of materials and medications purchased in non-government funded facilities;
		Inability to use community health insurance in private pharmacies
	Health services delivery related delays	
		High patient load and limited capacity of facilities

For peer review only

		Inadequate facility infrastructure
		Inadequate follow up of privately referred patients to the point of receiving care by referring physicians
		Reduced staff after daylight working hours
	Health workforce related delays	
		Insufficient skilled medical and specialist staff
		Unavailability of certain specialized services and the high cost of these services outside the country
		Poorly organized inter-facility referral system
		Traditional healers and their interface with the health system
	Health information related delays	
		Difficulty in the use of electronic medical systems by healthcare staff
	Medical products related delays	
		Unavailable equipment within facilities
		Unavailable medications or other treatment within facilities
**Fourth delay (delay in remaining care)**		
	Health services delivery related delays	
		Poor counseling of patient in need for rehabilitation by healthcare workers
		Previous unsatisfactory experience of health facility care
		Perceived early discharge in an unsatisfactory health condition
		Complications of alternative home-based care which may limit patients' movement
		Limited opening hours of some facilities
		Cumbersome referral system for gaining appointments and multiple transfers required for community health insurance users
		Lack of triage services at receptions; Slow service delivery at health centers
		Difficult follow-up process for discharged patients
	Health workforce related delays	
		Traditional healers counseling patients against returning for follow up
		Multi-tasking by healthcare workers and lack of staff supervision and many responsibilities on one staff in health centers
		Insufficiency of staff and decrease in confidence at HC
	Health information related delays	
		Use of paper-based health records

For peer review only

	Transport related delays	
		Lack of transportation for follow-up
		Difficult terrain between home and location for rehabilitation or physiotherapy

Delays in seeking care

A typical rural road accident scene would be crowded with bystanders observing the injured and discussing their presumed prognosis. Others at the site of injury would attempt to provide first aid, call the ambulance service and the police, reach out to any available family members using the injured individual's cell phone, manage traffic movements at the crash site, and obtain any information required by the police for post-accident legal procedures. The decision to move victims to a nearby health facility would usually be made after some (usually intense) discussion among bystanders. In the rural setting, the most proximate facilities are typically health centers and health posts and participants mentioned that only a small proportion of the injured would delay seeking care and return home for self-management or seek the services of a traditional healer.

At the scene of an accident, it was reported that in most cases, the initial decision to seek care was deferred by victims or bystanders on account of distance and time to health facilities from the accident location, lack of financial means including considerations of whether or not injured individuals had community health insurance and reticence about going through the process of activating community health insurance payments.

"*(...) when the patient doesn't have health insurance and doesn't have money, they fear going to health facilities as it is complicated. In Kigali city, it is even more complicated as you may*

For peer review only

go to the hospital and they ask you to pay around 50 to 60 thousand, and it is complicated. Then people may miss going to health facilities due to that." (FGDPatUrban01)

Furthermore, a poor understanding of the trauma victim's health condition and the need for care, coupled with an inaccurate perception of the severity of injury by the individual and by-standers, often delayed the decision to seek care. Occasionally, community health workers and village leaders would contribute to this delay if they felt the injury was not severe enough to necessitate care.

"… (..) *a case of a mother who had a child who was kicked against the wall by the sheep, and they said it was not serious, saying that it was normal for the children to fall and have minor pain, until the child continued to swell to the level that they decided to take him to the hospital very late." (FGDPatRural01)*

Socio-cultural and religious beliefs opposed to orthodox healthcare and prior positive experiences with traditional medicine use also contributed to this delay.

"What I also observed is that an individual may have an injury causing bleeding, and instead of taking him to the hospital, they choose to apply herbal medications and later go to the hospital with complications." (FGDPatRural01)."People's belief systems and confidence in traditional medicine contribute a lot to the decisions they take on what to do after having an accident." (FGDComUrban01)

In rural Rwanda, it is the community and family that often decide whether or not an injured member should receive care at a healthcare facility. This shared decision-making process may further delay seeking care. In cases of violent communal conflict, the community conflict management process also delays the decision to seek care, as settling the conflict is prioritized over taking decisions to take victims to a care facility.

For peer review only

"*In the community (…) the members of the wider community first come to see your husband or wife and the family then collectively decide to take you to the health facility*." *(FGDComRural01)*

Delays in reaching care

The injured individuals would usually be transported in traditional stretchers and on motorbikes, depending on their condition. In contrast, injured in urban Kigali would be typically carried by emergency services ambulances to district and referral hospitals within the city.

In the rural setting, they reported utilizing different modes of transportation from accident scenes to nearby health facilities. This ranged from walking on foot, riding commercial motorcycles, using traditional stretchers (see Figure 1), ambulances and renting private cars. The most common means were commercial motorcycles, if the condition of the injured permitted, and traditional stretchers carried by four members of the community were reserved for severely injured individuals in the rural setting.

Figure 1: A traditional stretcher.

Fig.1

Photo by Barnabas Alayande.

For peer review only

Only a few of the injured people reported reaching the health facilities by ambulances. The perception was that ambulances were scarce and were only used to transport pregnant women and children from home to hospital. Furthermore, in the rural areas, most people believed that the ambulance were only used for inter-facility transfer and not for initial transport to the hospital.

"I know that you have to first take your patient to the primary health facility, and if they decide to refer him, it is then the time they can call an ambulance." (FGDPatRural01)

This was in contrast to urban participants who reported the use of ambulances as the primary means to reach health facility. Alternatives in Kigali were only used when ambulances were not required, available or accessible.

"... I remember the last time an accident occurred, and there were so many accidents (....) Then there was the surprise of how they instructed the ambulance to deviate its initial direction to pass there and bring one medical personnel at the accident scene to return later after taking that first patient to the hospital." (FGDComUrban01)

Furthermore, the cost of private transport and the unwillingness of private drivers to assist in transporting the injured contributed to the delay in reaching the care. Private car owners, in particular, if they experienced complicated reception processes at health facilities in the past, were not willing to help transport the patients

(...) apart from having a human heart, people are hesitant to help in that situation because bringing the accident victim to the medical facility suggests that you are willing to spend your time there." (FGDComUrban01)

For peer review only

Additionally, high traffic density during rush hour in Kigali, the occurrence of simultaneous road traffic accidents, and COVID-19 curfew-related delays were cited.

“The most challenge is traffic jam, the ambulance may come on time but the road is busy with traffic jam.” (FGDPatUrban01) and *"during COVID-19, when there were curfew hours and you wanted to reach the hospital, the motorcycle rider would ask you for a lot of money, saying that he would face the traffic police and that he would need to use shortcut roads which are not safe. If you were lucky, he would take you to the hospital (…) [but] you had to pay a lot of money, almost three times the normal cost since you had no alternative.” (FGDPatRural01)*

The absence of personal insurance also led to delays in reaching care.

“… [a] patient who has personal financial challenges (…) advised me to first solve issues of community health insurance before going to the hospital to get care.” (PatRural002)

The inter-facility referral and transfer process also contributed to delays in receiving care, especially when it came to asking trauma victims to reach a higher-level facility unassisted and not in the ambulance.

"From the health center, they gave us a referral to [name of district hospital] without facility transport. Here, they also provided us with basic healthcare and a transfer to Ruhengeri hospital [referral hospital], but we had to do it ourselves. We needed four people to lift her [the injured patient] as she is old; it required me to take four motorcycles, one for her, one for me, and others for the ladies to care for her on each trip"(FGDPatRural01)

Delays in receiving care

Most of the participants in rural Burera first received care at the health post or the health center level, while those in Kigali City who used mostly emergency ambulance services to reach care,

For peer review only

commonly accessed higher levels of care through district hospitals and the University Teaching Hospital of Kigali (CHUK). However, across the board, the required medication and diagnostic equipment was not always available at these health facilities, and the prohibitive cost of trauma care materials and medications purchased outside of the government facilities delayed receiving care. Facility overload also emerged as a barrier, as hospitals had limited capacity to attend to several patients at once.

"(...) patients with multiple fractures come in and are laid on the ground. The hospital has a serious problem of shortage of beds and they do not have space to put all emergencies they receive." (FGDPatUrban01). "(...) the infrastructure we have doesn't meet the need as you may find, even the doctors are few. Patients they have to operate on don't have enough space for post-operative care, and this may limit services". (FGDComUrban01)

Some participants experienced delays in receiving care within the facility because of an insufficient number of skilled trauma care providers or specialists. One of the patients interviewed talked about experiencing a delay in receiving care because healthcare providers did not recognize the severity of their injuries.

"(...) they have low skills (...) like in case of fracture. You see, a general physician may not have enough skills on it, and you see them discussing it, and you hear them. They delayed doing this and you had to do this; you know, medical staff, the one who came first [to see me] was clearly working in slow-motion for unjustified reasons." (PatRural004)

Several participants mentioned the organization and flow of patients in the health facility process as being responsible for delays in receiving care. Specific barriers included gaps in the electronic medical records, a lengthy invoicing and record recovery system; difficulties in

For peer review only

effecting payments; inadequate communication by healthcare providers coupled with the patient's lack of familiarity with the health facility and long or short duration of treatment.

"*The patient should come and not be required to wait on those benches. They should go immediately to the cashier to pay and see their doctor. It would be better than sitting on those benches, getting a number before going on to pay. Furthermore, some people are assisted by security staff during the payment procedure. Even if you come first, you run the risk of going home unattended by the doctor, as they assist many people." (FGDPatUrban01)*

Another patient said *"I was discharged on Friday, but the process could not be completed that day, and some services were unavailable on Saturday and Sunday, so I had to wait until Monday. I believe they should create a shortcut so that the person who discharge you can use their technology to show the credit you have and pay directly." (PatUrban002)*

Additionally, *"People have a problem when they discharge them and no longer consider their situation.... you observe for a few days, they discharge you. They released me while both of my legs were stiff from the prolonged suspension." (FGDPatUrban01)*

Furthermore, issues surrounding insurance were cited by participants as causing delays at the facility. Patients with community health insurance reported that insurance staff were not always available to attend to patients during work hours, especially at the primary level of care.

"(...) I saw it in two facilities. You meet him [the insurance staff], and a short while later, when you return to see him, he is not at his workstation, and you have to wait while you are severely suffering. It is mandatory to see him as you cannot be attended to if the insurance worker does not help you." (PatUrban001)

For peer review only

Within public facilities, uninsured patients and those without financial means to purchase prescribed medication faced delays resulting from a relatively high direct cost of care and a long payment process that involved seeking hospital social and administrative approval to secure prescribed medication on credit. Outside government facilities, such as community pharmacies, health insurance was not accepted and the high cost of purchasing medications out of packet was a barrier to receiving care.

"If you have an accident and they have to put in the left leg prosthesis and they don't have it here, they send you to buy it for around 50,000 to 100,000 Rwandan Francs, while if you find it here, you pay only 10,000 Rwandan Francs [with community health insurance]." (FGDPatUrban01). "*Whenever they send you outside [of the health facility to private pharmacies], regardless of your insurance status, the amount of money billed for medication will be too much for you (FGDPatUrban01)*"

Delays to remaining in care

Most participants felt injured patients had been discharged before full recovery due to insufficient beds and the need to admit other trauma patients. This led to difficulties in returning for follow-up as the patient's condition was not optimal for the strain of the travel and the visit.

"*Often, the patient is discharged before achieving total recovery (...) the doctor decides to continue following up with the patient while at home. In that situation, the patient may not be satisfied." (FGDComUrban01)*

"(...) the long travel on foot made it swollen, and I reached there with the arm all swollen. I had to be there early, an hour and a half before any physical examination, so that the swelling would go down and the doctor would not be confused. I don't want him to think that I have

For peer review only

another problem while the reason for the swelling was actually from traveling for the appointment." (PatUrban010)

While agreeing that they had been given follow-up and rehabilitation appointments, the distance from home to rehabilitation facilities, difficulties in arranging transport, and the cost of transport were common barriers to returning. Some injured patients visited traditional healers and were subsequently dissuaded from attending the follow-up appointments in the health facility or hospital.

Most of the interviewed participants were community health insurance users. They identified delays caused by the need for patients to present a new transfer from lower levels of care before being attended to for follow-up at higher levels of care from which they were discharged. Presenting for rehabilitation or follow-up at a tertiary facility necessitates returning to the health center and district hospital to ensure insurance claim payment by obtaining a new referral or transfer for each visit that exceeds 30 days following discharge.

"When I was discharged, I came back [for my follow-up appointment] and stayed there till 3:00 pm. When I finally reached those cashiers, they asked me for the transfer, and I said I had an appointment. They insisted I needed the transfers. I asked them why because I was previously admitted, and they told me to go back to the health center and then to the district hospital to get the transfers and to reschedule my appointment for later. I (…) finally went back home, then to the health center and from there to the district hospital before finally coming back again to ask for a new appointment." (FGDPatUrban01)

DISCUSSION

This study is the first qualitative study, of which we are aware, to identify barriers to injury care from the point of injury to rehabilitation in both rural and urban settings in Rwanda,

For peer review only

utilizing the four delays framework. A recent systematic review found that very few studies in lower- and middle-income countries have comprehensively assessed barriers to access to quality trauma care (8) from being injured to being discharged from care. What is more, we focused on urban and rural settings in recognition of possible disparities that may exist in accessing injury care in Rwanda. Multiple barriers to injury care in were identified across the four delays, in both rural and urban settings. The barriers most mentioned by patients and community leaders were "insufficient number of competent trauma care providers," “rigid referral process," and "facility-related delays in delivery of care." These findings echo the results of a multidisciplinary stakeholder consultation aimed at mapping injury care access in Rwanda, which found that "training and retention of specialist staff," "geographical coverage of referral trauma centers," and "lack of protocol for a bypass to referral centers" among the top 4 barriers identified by the participants (9). The similarity of findings from these two studies, despite the differing perspectives of contributors, suggests that interventions aimed at ensuring access to safe, timely, affordable and quality injury care should take into consideration these specific barriers.

Regarding transport for injured patients to facilities that can manage their injuries, a limited number of ambulances available to convey injured patients from the point of injury to the appropriate trauma care facility was identified as a cause of delay. A systematic review of literature in LMICs found that often ambulances were not available, but even when ambulances are available, many emergency and trauma patients are forced to use alternative means of transport (14). Rwanda is relatively unusual in sub-Saharan Africa in having a country-wide ambulance service. However, discussions at the aforementioned workshop and in the 2019 Rwanda trauma symposium, highlighted that efficiency could be improved with better location of the patient and communication between the ambulances and facilities (9)(15). Studies are ongoing to address these issues(16)(17). Although our participants in Kigali stated that injured

For peer review only

people often used ambulances, it was clear that their availability was much lower in rural areas. Rwanda's unique hilly topography must be considered when implementing low-cost and innovative ambulance services for rural populations. Another perspective, however, may be that participants did not know how to access the ambulance service. The widespread perception in this study was that in rural areas, ambulance use was restricted to only transporting pregnant women or sick children from home to hospital. Rwanda's Service d'Aide Medicale d'Urgence (SAMU) was founded in 2008 and now has up to 225 ambulances (five per district). This results in an ambulance to population ratio of 1:48,000 and exceeds the World Health Organization (WHO) recommendation of 1:50,000 (14). The rural-urban disparity in the perception of the availability of ambulances for injury care should be addressed. Community education, alongside improved coordination and logistics of ambulance distribution for injury care, should to be optimized (18) (19)

In Sub-Saharan Africa, surgical and anesthetist densities are generally low. By 2015, Rwanda had only 0.79 per 100,000 skilled health workers, a significantly lower ratio than in High-Income Countries HICs (20). Trauma care providers are perceived to be in short supply and inequitably distributed in favor of urban areas. The participants in this study identified "insufficient number of qualified trauma care providers" as a major barrier to receiving injury care. Hutch et al. found that only 20 surgeons were trained by the College of Surgeons for East Central and Southern Africa in the 13 years leading to 2013(21). Despite this low number of surgeons, Rwanda has a high level of surgeon retention (90%) following training (21). However, this retention is particularly skewed towards urban areas, and an attrition rate of up to 80% for doctors in the rural setting has been identified (22). Rwandan anesthesiologists and surgeons with specialized training work almost entirely in urban referral hospitals, leaving surgical care at rural district hospitals largely to general practice physicians and nurses (23). The presence of trained anesthetists and the availability of anesthesia are essential to operative

For peer review only

injury care. A 2017 study found that over a third of anesthetists trained in Rwanda had emigrated for a variety of reasons, ranging from a desire for better remuneration to a lack of equipment and medication for safe anesthesia, occupational isolation in rural settings, and demoralization (24). Only about 75 physiotherapists serve Rwanda's entire population of over 13 million people (25). This issue of insufficient trauma care providers is in keeping with findings from other LMICs. In a systematic review of barriers to out-of-hospital emergency trauma care, over 60% of them identified a lack of skilled personnel as a key barrier (14); lack of available, trained, and motivated clinical staff was the second highest barrier to access to quality trauma care identified in an international Delphi exercise(19). The WHO recognizes the role of various levels of healthcare delivery in trauma management. Non-physician providers and non-specialist providers (general practitioners) in low-income countries are responsible for a large portion of trauma care (26). Therefore, human resource development for injury care and appropriate staffing should be prioritized at all levels of healthcare delivery alongside the provision of required infrastructure, equipment, and supplies to facilitate the better delivery of trauma care (26). Models such as Rwanda's Human Resource for Health program should be improved upon and strengthened to optimize trauma care specialist training (27). Training and retention of surgeons and anesthetists, particularly in rural areas, should be encouraged and incentivized (28).

For peer review only

Rwanda has one of the most robust health insurance population coverages in sub-Saharan Africa, with community-based health insurance held by over 91% of individuals(29). This facilitates much needed access to care for those who have insurance. However, it is not yet comprehensive enough to benefit all injured patients (9)(30)(31). Participants in both interviews and focus group discussions mentioned barriers related to community health insurance. Multiple insurance-related referral processes, long payment procedures within public health facilities for insurance holders, and non-acceptance in private pharmacies

contributed to the delays. Additionally, procedures to prove possession of insurance meant that many patients in our study experienced further delays. The dissatisfaction with the procedures for checking insurance has been cited as one of the major barriers to community-based health insurance renewal (32), despite the fact that 97% of those in the scheme say they benefit from it (30). For the community health insurance to have a strong positive impact on access to healthcare, those limitations have to be addressed (31), and more efforts should be engaged toward the development of prehospital and inter-facility transportation arrangements, strengthening district hospitals, and better supporting referral institutions (33).

Many participants in this study reported being discharged before they perceived that they were fully recovered. They believed that the early discharge was due to insufficient bed space. Early mobilization and discharge maybe advantageous for trauma patients to reduce complications of deep vein thrombosis, joint stiffness, and depression, among others (34). However, this early discharge was not complemented by the availability of decentralized rehabilitation services. To be able to return to the treating tertiary facility for physiotherapy or other rehabilitation services for appointments beyond the 30 days allowed by community health insurance, the patients in our study were asked to go through the entire referral process again, without which their insurance payments would not be guaranteed. This involved returning to the initial referral health center, then to the next district facility that had referred the patient, and finally to the tertiary facility with appointments. This time-consuming procedure was viewed as a barrier to continuing in care.

At the University Teaching Hospital of Kigali, up to 84% of orthopedic trauma patients (most of whom required post-discharge rehabilitation) have been found to come from facilities in other provinces rather than Kigali. This pattern results in a heightened need for inter-facility transfer (33). Our study suggests that a simplified pathway to follow-up care for injury patients

For peer review only

may help reduce losses to follow-up. In addition, options for home-based primary care and other innovative methods may need to be considered and tested (35), based on the needs and preferences of the communities. These reflections align with other studies. For example, using an online questionnaire distributed through a variety of international and regional organizations from the various WHO regions, Darci et al. found that 31.8% of respondents viewed providing rehabilitation services within specialized hospitals and units completely unacceptable, relative to general hospitals or non-specialized units. Up to 79.3% of the same respondents preferred community-based rehabilitation over hospital-based rehabilitation (36).

This study is not without its limitations. Whilst Kigali is the major conurbation in Rwanda, it is not the only one and our findings from Kigali may not be representative of other urban settings. Likewise, the rural interviews were conducted only in Burera, and certain nuances of injury care access within rural Rwanda may have been missed because of the wide variation in rural settlements while we recognize that barriers may differ slightly by district setting. Additionally, most (75%) of the patients were male, whilst this may mean that we missed barriers mostly experienced by females, it should be noted that the majority of injured patients in Rwanda are male.

CONCLUSION

In contrast to previous studies that focused primarily on the delay to receiving care in health facilities, this qualitative study provides insights into barriers to seeking, reaching, receiving and remaining in care from the injured patients', caregivers' and community leaders' perspectives. In both rural and urban settings, hindrances along the spectrum of care, from injury to rehabilitation, and at various levels of the health system, have been identified. These barriers should be considered and guide injury interventions and health facility organization. Human resources for health strengthening specific to injury care, facility re-organization to

For peer review only

reduce in-facility waiting times, and revision of the complex follow-up referral system are priority areas for intervention. Future health policy planning should strengthen trauma care by focusing and addressing these barriers.

Ethics approval

The overall study was approved by University of Birmingham Research Ethics Committee, UK (ERN_20-00880). Data collection was approved by the National Health Research Committee (NHRC/2020/PROT/044) for Rwanda. Additional approval was sought from the respective hospitals before visiting the facilities.

Author contributions

JCB and AB led the study in Rwanda; JID and JCB conceived the concept for the study; PN collected data; PN, BA, MLO, AI and other authors contributed to the analysis of results; PN and BA wrote the initial draft of the manuscript, and all other authors brought their inputs into the draft manuscript and accepted the final version. JCB, the senior author, accepts full responsibility for the conduct of this study. All authors agree to be accountable for all aspects of the work.

Data sharing statement

The data that support the findings of this study are available upon reasonable request from the corresponding author. The data are not publicly available due to restrictions as they contain information that could compromise the privacy of research participants.

Competing interest: None declared

Word count:

For peer review only

REFERENCES

1. Anon. Global burden of 369 diseases and injuries in 204 countries and territories, 1990-2019: a systematic analysis for the Global Burden of Disease Study 2019. Lancet (London, England) 2020;396:1204–1222.

2. Obermeyer Z, Abujaber S, Makar M, et al. Emergency care in 59 low- and middle-income countries: A systematic review. Bull World Health Organ World Health Organization 2015;93:577–586.

3. The World Bank. Cause of death, by injury (% of total) - Rwanda. Data 2020. Available at: https://data.worldbank.org/indicator/SH.DTH.INJR.ZS?locations=RW. Accessed June 23, 2022.

4. Kim WC, Byiringiro JC, Ntakiyiruta G, et al. Vital Statistics: Estimating Injury Mortality in Kigali, Rwanda. World J Surg 2016;40:6–13.

5. Edem IJ, Dare AJ, Byass P, et al. External injuries, trauma and avoidable deaths in Agincourt, South Africa: a retrospective observational and qualitative study. BMJ Open 2019;9:e027576.

6. Stewart B, Hollis S, Amato SS, et al. Trauma care and development assistance: Opportunities to reduce the burden of injury and strengthen health systems. Bull World Health Organ 2019;97:371–373.

7. Calvello EJ, Skog AP, Tenner AG, Wallis LA. Applying the lessons of maternal mortality reduction to global emergency health. Bull World Health Organ 2015;93:417–423.

8. Whitaker J, O'Donohoe N, Denning M, et al. Assessing trauma care systems in low-

For peer review only

income and middle-income countries: A systematic review and evidence synthesis mapping the Three Delays framework to injury health system assessments. BMJ Glob Heal 2021;6:1–15.

9. Odland Maria Lisa, Dmitri, Achieng, Nepogodiev John C, et al. Identifying , Prioritizing and Visually Mapping Barriers to Injury Care in Rwanda : A Multi-disciplinary Stakeholder Exercise. World J Surg 2020;44:2903–2918.

10. Rosenberg A, Ntirenganya F, Bagahirwa I, et al. First Rwanda National Trauma Symposium 2019: Challenges and priorities. J Glob Health. 2020;10(1):010201. doi:10.7189/jogh.10.010201

11. Odland ML, Whitaker J, Nepogodiev D, et al. Identifying, Prioritizing and Visually Mapping Barriers to Injury Care in Rwanda: A Multi-disciplinary Stakeholder Exercise [published correction appears in World J Surg. 2022 Jul;46(7):1805]. World J Surg. 2020;44(9):2903-2918. doi:10.1007/s00268-020-05571-6

12. Rwanda Ministry of Health. Fourth Health Sector Strategic Plan. Ministry of Health. 2018.

13. Lyn Richards. Handling Qualitative Data: A Practical Guide. Sage Publ. xii:207. Available at: https://www.qualitative-research.net/index.php/fqs/article/view/107/223. Accessed June 16, 2022.

14. Kironji AG, Hodkinson P, De Ramirez SS, et al. Identifying barriers for out of hospital emergency care in low and low-middle income countries: A systematic review. BMC Health Serv Res 2018;18:1–20.

15. Rosenberg A, Ntirenganya F, Bagahirwa I, et al. First Rwanda National Trauma Symposium 2019: Challenges and priorities. J Glob Health 2020;10:1–8.

16. Ashley Rosenberga, Rob Rickardb, Fraterne Z. Uwinshutic, Gabin Mbanjumucyod, Menelas Nkeshimanad, JeanMarie Uwitonzec, Ignace Kabagemac, Theophile Dushimec SJ.

For peer review only

Collaboration for preliminary design of a mobile health solution for ambulance dispatch in Rwanda. 2020.

17. Jayaraman S, Ntirenganya F, Nkeshimana M, et al. Building trauma and ems systems capacity in rwanda: Lessons and recommendations. Ann Glob Heal 2021;87:1–13.

18. Kannan VC, Tenner A, Sawe HR, et al. Emergency care systems in Africa: A focus on quality. African J Emerg Med 2020;10:S65–S72.

19. Whitaker J, Nepogodiev D, Leather A, Davies J. Assessing barriers to quality trauma care in low and middle-income countries: A Delphi study. Injury Netherlands 2020;51:278–285.

20. Ministry of Health. Health Labor Market Analysis Report. 2019.

21. Hutch A, Bekele A, O'Flynn E, et al. The Brain Drain Myth: Retention of Specialist Surgical Graduates in East, Central and Southern Africa, 1974–2013. World J Surg 2017;41:3046–3053.

22. Odhiambo J, Rwabukwisi FC, Rusangwa C, et al. Health worker attrition at a rural district hospital in Rwanda: A need for improved placement and retention strategies. Pan Afr Med J 2017;27:1–7.

23. Notrica MR, Evans FM, Knowlton LM, Kelly McQueen KA. Rwandan surgical and anesthesia infrastructure: A survey of district hospitals. World J Surg 2011;35:1770–1780.

24. Skelton T, Irakoze A, Bould MD, et al. Retention and Migration of Rwandan Anesthesiologists: A Qualitative Study. Anesth Analg 2020;131:605–612.

25. World Physiotherapy. Rwanda Physical Therapy Organisation. Rwanda profile 2020. Available at: https://world.physio/membership/rwanda. Accessed February 24, 2022.

26. Dries DJ. Guidelines for Essential Trauma Care. Shock 2005;23:97–98.

27. Binagwaho A, Kyamanywa P, Farmer PE, et al. The Human Resources for Health Program in Rwanda — A New Partnership. N Engl J Med 2013;369:2054–2059.

28. Van Essen C, Steffes BC, Thelander K, et al. Increasing and Retaining African Surgeons

For peer review only

Working in Rural Hospitals: An Analysis of PAACS Surgeons with Twenty-Year Program Follow-Up. World J Surg 2019;43:75–86.

29. Kalisa, Musange, David S& K. The Development of Community-Based Health Insurance in Rwanda: Experiences and Lessons. Univ Rwanda, msh, Rockefeller Found 2016;1.

30. David Collins US. The Impact of Community-Based Health Insurance on Access to Care and Equity in Rwanda BACKGROUND. Manag Sci Heal MSH 2016:1–8.

31. Saksena P, Antunes AF, Xu K, et al. Mutual health insurance in Rwanda: Evidence on access to care and financial risk protection. Health Policy (New York) 2011;99:203–209.

32. Hussien M, Azage M. Barriers and facilitators of community-based health insurance policy renewal in low-and middle-income countries: A systematic review. Clin Outcomes Res 2021;13:359–375.

33. Ntakiyiruta G, Wong EG, Rousseau MC, et al. Trauma care and referral patterns in Rwanda: Implications for trauma system development. Can J Surg 2016;59:35–41.

34. Kang Y, Liu J, Chen H, et al. Enhanced recovery after surgery (ERAS) in elective intertrochanteric fracture patients result in reduced length of hospital stay (LOS) without compromising functional outcome. J Orthop Surg Res 2019;14:1–7.

35. Rezaei M, Sharifi A, Vaccaro A, Rahimi-Movaghar V. Home-based rehabilitation programs: Promising field to maximize function of patients with traumatic spinal cord injury. Asian J Neurosurg 2019;14:634.

36. Darzi AJ, Officer A, Abualghaib O, Akl EA. Stakeholders' perceptions of rehabilitation services for individuals living with disability: A survey study. Health Qual Life Outcomes 2016;14:1–9.

For peer review only

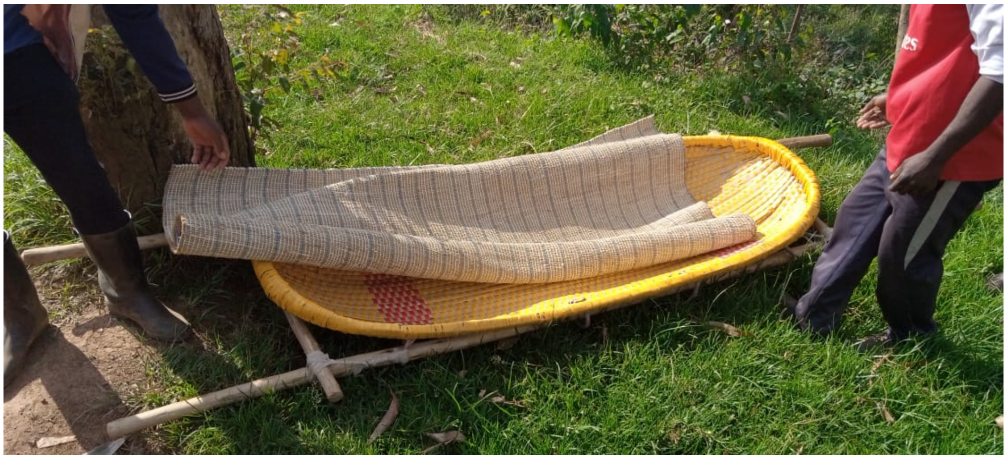

A Rwandan traditional stretcher

28448x12801mm (1 x 1 DPI)

Standards for Reporting Qualitative Research (SRQR)*

http://www.equator-network.org/reporting-guidelines/srqr/

Title and abstract

	Page/line no(s).
**Title** - Concise description of the nature and topic of the study Identifying the study as qualitative or indicating the approach (e.g., ethnography, grounded theory) or data collection methods (e.g., interview, focus group) is recommended	Page 1/lines 1-3
**Abstract** - Summary of key elements of the study using the abstract format of the intended publication; typically includes background, purpose, methods, results, and conclusions	Pages 2 and 3

Introduction

**Problem formulation** - Description and significance of the problem/phenomenon studied; review of relevant theory and empirical work; problem statement	Page 4
**Purpose or research question** - Purpose of the study and specific objectives or questions	Page 5/Lines 15-18

Methods

**Qualitative approach and research paradigm** - Qualitative approach (e.g., ethnography, grounded theory, case study, phenomenology, narrative research) and guiding theory if appropriate; identifying the research paradigm (e.g., postpositivist, constructivist/ interpretivist) is also recommended; rationale**	Page 7/Lines 14-16
**Researcher characteristics and reflexivity** - Researchers' characteristics that may influence the research, including personal attributes, qualifications/experience, relationship with participants, assumptions, and/or presuppositions; potential or actual interaction between researchers' characteristics and the research questions, approach, methods, results, and/or transferability	Pages 6 and 7
**Context** - Setting/site and salient contextual factors; rationale**	Page 7/Lines 16-21
**Sampling strategy** - How and why research participants, documents, or events were selected; criteria for deciding when no further sampling was necessary (e.g., sampling saturation); rationale**	Page 8/Lines 1-15
**Ethical issues pertaining to human subjects** - Documentation of approval by an appropriate ethics review board and participant consent, or explanation for lack thereof; other confidentiality and data security issues	Page 27/Lines 8-12<br>Page 8/Lines 11-14
**Data collection methods** - Types of data collected; details of data collection procedures including (as appropriate) start and stop dates of data collection and analysis, iterative process, triangulation of sources/methods, and modification of procedures in response to evolving study findings; rationale**	Page 8/Lines 1-19

For peer review only - http://bmjopen.bmj.com/site/about/guidelines.xhtml

**Data collection instruments and technologies** - Description of instruments (e.g., interview guides, questionnaires) and devices (e.g., audio recorders) used for data collection; if/how the instrument(s) changed over the course of the study	Page 7/Lines 21-22
**Units of study** - Number and relevant characteristics of participants, documents, or events included in the study; level of participation (could be reported in results)	Page 9/Lines 18-21
**Data processing** - Methods for processing data prior to and during analysis, including transcription, data entry, data management and security, verification of data integrity, data coding, and anonymization/de-identification of excerpts	Page 8/Lines 16-24.<br>Page 9/Lines 1-14
**Data analysis** - Process by which inferences, themes, etc., were identified and developed, including the researchers involved in data analysis; usually references a specific paradigm or approach; rationale**	Page 8/Lines 16-24.<br>Page 9/Lines 1-14
**Techniques to enhance trustworthiness** - Techniques to enhance trustworthiness and credibility of data analysis (e.g., member checking, audit trail, triangulation); rationale**	Page 9/Lines 1-14

Results/findings

**Synthesis and interpretation** - Main findings (e.g., interpretations, inferences, and themes); might include development of a theory or model, or integration with prior research or theory	See table 1: Page 10
**Links to empirical data** - Evidence (e.g., quotes, field notes, text excerpts, photographs) to substantiate analytic findings	Pages 13-21

Discussion

**Integration with prior work, implications, transferability, and contribution(s) to the field -** Short summary of main findings; explanation of how findings and conclusions connect to, support, elaborate on, or challenge conclusions of earlier scholarship; discussion of scope of application/generalizability; identification of unique contribution(s) to scholarship in a discipline or field	Pages 22-26
**Limitations** - Trustworthiness and limitations of findings	Page 26 Lines 13-20

Other

**Conflicts of interest** - Potential sources of influence or perceived influence on study conduct and conclusions; how these were managed	Page 5/Lines 4 and 5
**Funding** - Sources of funding and other support; role of funders in data collection, interpretation, and reporting	Page 5/Lines 1-3

*The authors created the SRQR by searching the literature to identify guidelines, reporting standards, and critical appraisal criteria for qualitative research; reviewing the reference lists of retrieved sources; and contacting experts to gain feedback. The SRQR aims to improve the transparency of all aspects of qualitative research by providing clear standards for reporting qualitative research.

For peer review only

For peer review only - http://bmjopen.bmj.com/site/about/guidelines.xhtml

**The rationale should briefly discuss the justification for choosing that theory, approach, method, or technique rather than other options available, the assumptions and limitations implicit in those choices, and how those choices influence study conclusions and transferability. As appropriate, the rationale for several items might be discussed together.

Reference:

O'Brien BC, Harris IB, Beckman TJ, Reed DA, Cook DA. **Standards for reporting qualitative research: a synthesis of recommendations.** *Academic Medicine*, Vol. 89, No. 9 / Sept 2014 DOI: 10.1097/ACM.0000000000000388

For peer review only

For peer review only - http://bmjopen.bmj.com/site/about/guidelines.xhtml

BMJ Open

Barriers to equitable access to quality trauma care in Rwanda: a qualitative study

Journal:	*BMJ Open*
Manuscript ID	bmjopen-2023-075117.R1
Article Type:	Original research
Date Submitted by the Author:	04-Aug-2023
Complete List of Authors:	Nzasabimana, Pascal; University of Rwanda, Single Project Implementation Unit<br>Ignatowicz, Agnieszka ; University of Birmingham, Institute of Applied Health Research<br>Alayande, Barnabas; University of Global Health Equity, Center for Equity in Global Surgery<br>Abdul-Latif , Abdul-Malik ; University of Birmingham, Institute of Applied Health Research<br>Odland, Maria Lisa; University of Birmingham, Institute of Applied Health Research; St Olav's Hospital, Trondheim University Hospital, Department of Obstetrics and Gynecology<br>Davies, Justine ; University of Birmingham, Institute of Applied Health Research; Stellenbosch University, Department of Global Health, Centre for Global Surgery<br>Bekele, Abebe; University of Global Health Equity, Center for Equity in Global Surgery<br>BYIRINGIRO, JEAN CLAUDE; University of Rwanda College of Medicine and Health Sciences, School of Medicine and Pharmacy
**Primary Subject Heading**:	Health services research
Secondary Subject Heading:	Health services research, Public health, Surgery
Keywords:	Health Services Accessibility, Orthopaedic & trauma surgery < SURGERY, Organisation of health services < HEALTH SERVICES ADMINISTRATION & MANAGEMENT

SCHOLARONE™
Manuscripts

I, the Submitting Author has the right to grant and does grant on behalf of all authors of the Work (as defined in the below author licence), an exclusive licence and/or a non-exclusive licence for contributions from authors who are: i) UK Crown employees; ii) where BMJ has agreed a CC-BY licence shall apply, and/or iii) in accordance with the terms applicable for US Federal Government officers or employees acting as part of their official duties; on a worldwide, perpetual, irrevocable, royalty-free basis to BMJ Publishing Group Ltd ("BMJ") its licensees and where the relevant Journal is co-owned by BMJ to the co-owners of the Journal, to publish the Work in this journal and any other BMJ products and to exploit all rights, as set out in our licence.

The Submitting Author accepts and understands that any supply made under these terms is made by BMJ to the Submitting Author unless you are acting as an employee on behalf of your employer or a postgraduate student of an affiliated institution which is paying any applicable article publishing charge ("APC") for Open Access articles. Where the Submitting Author wishes to make the Work available on an Open Access basis (and intends to pay the relevant APC), the terms of reuse of such Open Access shall be governed by a Creative Commons licence – details of these licences and which Creative Commons licence will apply to this Work are set out in our licence referred to above.

Other than as permitted in any relevant BMJ Author's Self Archiving Policies, I confirm this Work has not been accepted for publication elsewhere, is not being considered for publication elsewhere and does not duplicate material already published. I confirm all authors consent to publication of this Work and authorise the granting of this licence.

For peer review only

Barriers to equitable access to quality trauma care in Rwanda: a qualitative study

Pascal Nzasabimana[1], Agnieszka Ignatowicz[2], Barnabas Alayande[3,4], Abdul-Malik Abdul-Latif[2], Maria Lisa Odland[2,8,9,10], Justine I Davies[2,5,6*], Abebe Bekele[3*], Jean Claude Byiringiro[7*].

1. Single Project Implementation Unit, University of Rwanda, Kigali, Rwanda
2. Institute of Applied Health Research, University of Birmingham, Birmingham, UK
3. Center for Equity in Global Surgery, University of Global Health Equity, Kigali, Rwanda
4. Program in Global Surgery and Social Change, Harvard Medical School, Boston, USA
5. Centre for Global Surgery, Department of Global Health, Stellenbosch University, Cape Town, South Africa
6. Medical Research Council/Wits University Rural Public Health and Health Transitions Research Unit, Faculty of Health Sciences, School of Public Health, University of the Witwatersrand, Johannesburg, South Africa
7. School of Medicine and Pharmacy, University of Rwanda, Kigali, Rwanda
8. Department of Obstetrics and Gynecology, St Olav's Hospital, Trondheim University Hospital, Trondheim, Norway
9. Malawi-Liverpool-Wellcome Trust Research Institute, Blantyre, Malawi
10. Institute of Life Course and Medical Sciences, University of Liverpool, Liverpool, United Kingdom

Correspondence to Dr Agnieszka Ignatowicz, Institute of Applied Health Research, University of Birmingham, Birmingham, B15 2TT, United Kingdom. Email: a.m.ignatowicz@bham.ac.uk

For peer review only

*Co-senior authors

Abstract

Objectives: Using the "Four Delay" framework, our study aimed to identify and explore barriers to accessing quality injury care from the injured patients', caregivers', and community leaders' perspectives.

Design: A qualitative study assessing barriers to trauma care comprising twenty in-depth semi-structured interviews and four focus group discussions were conducted. The data were analyzed thematically.

Setting: This qualitative study was conducted in Rwanda's rural Burera District, located in the Northern Province, and in Kigali City, the country's urban capital, to capture both the rural and urban population's experiences of being injured.

Participants: Purposively selected participants were individuals from urban and rural communities who had accessed injury care in the previous six months or cared for the injured people, and community leaders. Fifty-one participants, 13 females and 38 males ranging from 21 to 68 years of age participated in interviews and focus group discussions. Thirty-six (71%) were former trauma patients with a wide range of injuries including fractured long bones (9, 45%), other fractures, head injury, polytrauma (3, 15% each), abdominal trauma (1, 5%), and lacerations (1, 5%), while the rest were caregivers and community leaders.

Results: Multiple barriers were identified cutting across all levels of the "Four Delays" framework, including barriers to seeking, reaching, receiving, and remaining in care. Key barriers mentioned by participants in both interviews and focus group discussions were: lack of community health insurance, limited access to ambulances, insufficient number of trauma

For peer review only

care specialists, and a high volume of trauma patients. The rigid referral process and lack of decentralized rehabilitation services were also identified as significant barriers to accessing quality care for injured patients.

Conclusions: Future interventions to improve access to injury care in Rwanda must be informed by the identified barriers along the spectrum of care, from the point of injury to receipt of care and rehabilitation.

Keywords: Health Services Accessibility, Wounds and injuries, Rwanda

Strengths and limitations of this study:

- The study assessed access to trauma care access using a comprehensive "Four Delay" framework that also emphasized remaining in care.
- A multi-stakeholder perspective on delay is obtained by triangulating the perspectives of the service user, caregiver, and community leader.
- Individual in-depth interviews and focus group discussions in the local language were carried out on a diverse group of participants from Rwanda's urban and rural communities.
- All transcripts of interviews and focus group discussions were coded in parallel by a context and local language fluent member as well as qualitative data experts to ensure rigor.
- 75% of study participants are male, and even though the majority of injured patients in Rwanda are male, we recognize that this may limit the generalizability of results.

Funding Statement

For peer review only

Funding for this study was provided by the UK National Institute of Health Research (NIHR), award number 130036. The views expressed in this publication are those of the author(s) and not necessarily those of the NIHR or the UK government.

Competing interests statement

None declared.

INTRODUCTION

Historically, global health initiatives have prioritized infectious diseases. However, in 2019 alone, the world recorded 4.3 million deaths from trauma, more deaths than malaria, tuberculosis, and AIDS combined (1). This burden is heaviest in Low- and Middle- Income Countries (LMICs), where injury rates are high, trauma systems are underdeveloped, and patients requiring emergency care are young and economically active (2). Up to 11% of deaths in Rwanda and 22% of deaths in the largest urban city of Kigali, housing 1,135,428 people according to the 2012 population census, are due to injury, with the most common cause being road traffic collisions (3)(4). Although injury prevention initiatives are necessary, identifying health system deficiencies and barriers that hinder access to safe, affordable, good quality and timely trauma care is key to reducing preventable deaths from people who are inevitably injured. A South African study suggests that strengthening health systems by focusing on inter-facility transfer systems would reduce up to 59% of preventable trauma deaths resulting from delays in receiving care. In addition, the same study argues that 23% and 18% of deaths from injury can be prevented by mitigating delays in seeking and reaching trauma care, respectively (5). Identifying and characterizing specific barriers to care is crucial for developing and implementing successful interventions, and reducing the burden of injury (6).

For peer review only

The "Three Delay" framework was developed originally to identify and mitigate barriers to maternal and child care but has been successfully applied to emergency trauma care and injury care (7). A systematic review and evidence synthesis mapping this framework to injury health system assessments found that approximately 79% of studies focused on delays within facilities, 46% assessed delays in reaching care, and only 10% assessed delays in seeking care (8). Research from sub-Saharan Africa comprised 40.1% of the studies assessed in this systematic review. A more comprehensive "Four Delay" framework for assessing trauma care access has been suggested, comprising delays in seeking, reaching, receiving, and remaining in care (9). This more comprehensive model emphasizes the need for holistic care, post-discharge physiotherapy, and other rehabilitation for the injured patient. In a workshop carried out to map barriers to trauma care in Rwanda using the four-delay framework, health providers, academia and government representatives identified 19 high-priority barriers faced by trauma patients and prioritized the top four. Three of the prioritized four were related to receiving care, but most of the barriers cut across multiple components of the four-delay model (9). However, the perspectives of the injured patients in the Rwandan context have not yet been explored. Our study focused on identifying and exploring barriers to accessing quality care from the perspective of the injured patients to develop priorities for trauma system interventions in Rwanda.

METHODS

This study was conducted using a grounded theory qualitative approach and a constructivist research paradigm. The Standards for Reporting Qualitative Research (SRQR) checklist guided all components of the writing and reporting of this study. This study was conducted concurrently with studies in Ghana and South Africa as part of the Equitable access to quality

For peer review only

trauma systems in Lower and Middle-Income Countries: A qualitative study assessing gaps and developing priorities (10).

Patient and Public Involvement Statement

This study was preceded by a National Trauma Symposium in 2019 organized by the Rwanda Ministry of Health and partners, where over 100 community stakeholders including representatives of the health ministry, Rwanda National Police, local non-governmental organizations, universities, emergency respondents, students, and medical practitioners identified access to trauma care as a national research priority (10). This work was also premised on a two-day workshop held in Kigali, Rwanda in May 2019 with representation from community, academia, and government which aimed to map barriers to accessing care from injury to rehabilitation and is in line with public priorities (11,12). Injured patients, caregivers and other stakeholders were involved in the design and execution of the study.

Researcher characteristics and reflexivity

Four of the eight authors live and work in Rwanda and served as context experts. Of these four, three are surgical providers who provide various degrees of trauma care and leadership to the trauma system. Overall leadership for the work in Rwanda was provided by a Rwandan trauma surgeon. Interviews were conducted by an investigator who is from rural Rwanda, speaks Kinyarwanda, and has lived experience of the health system and delays. There was adequate gender balance on the research team. Two UK-based investigators are qualitative method experts. The team is experienced in such studies as seven of the eight investigators were involved in similar surgical systems assessments in Ghana and South Africa.

Context

For peer review only

The study was conducted in Rwanda's rural Burera District, located in the Northern Province, and in Kigali City, the country's urban capital. Kigali City has a population of approximately 1,135,428 people concentrated on 1,760/km^2, whereas Burera district has a population of 336,455 people distributed over 522/km^2 (11). Burera, in terms of population and infrastructure, is a typical rural Rwandan district. Rwanda's urbanization is dominated by Kigali, which houses nearly 60% of the urban population and continues to experience rapid population and economic growth while modernizing and upgrading its infrastructure and transportation systems (12). Road traffic injuries are a major cause of trauma deaths in Rwanda, particularly among young men (13). Injury care pathways generally start with community health workers who are embedded in the communities, who refer to local health centers, and subsequently to second tier district hospitals, who refer to tertiary teaching hospitals and eventually to the country's single quaternary health institution. Many health facilities have ambulances that can transport injured patients to higher levels of care. The country's terrain is largely hilly. Most Rwandans (over 90%) are covered by *Mutuelle de Santé*, Rwanda's elaborated Community-Based Health Insurance program.

Sampling strategy

Using purposive sampling, participants of the twenty in-depth interviews and two focus group discussions were drawn from the hospital register if they were over the age of 18 and had either received injury care themselves within the previous six months or had cared for trauma victims who had received care. Local religious leaders, business leaders, service provider leadership, traditional healers, and local government authority figures participated in two community leaders' focus group discussions. Individuals under the age of 18 or with any intellectual impairments or cognitive deficit that would limit informed consent and participation, as well as service users accessing acute care during the study period, were

For peer review only

excluded. Individuals who returned for follow up and successfully remained in care however were not excluded.

Data collection methods

A list of patients treated for traumatic injuries in the last six months was obtained using information obtained from hospital admission registers at the emergency department and surgical wards of Centre Hospitalier Universitaire de Kigali (CHUK), Rwanda's leading center for tertiary trauma care provision, and Butaro District Hospital, a district facility in northern Rwanda. Patient caregivers identified through the trauma patients also took part in the study. A list of community leaders within the facilities' catchment area was also obtained through the administration departments of both hospitals.

Participants were oriented to the study by the interviewer (PN) who explained the aims and objectives of the study to participants in Kinyarwanda, ensuring verbal feedback from potential participants to confirm that they understood the study, and their potential role. Written informed consent was subsequently obtained. Individual interviews lasted 30 minutes on average, and focus groups discussions lasted between one and one and a half hours for six to nine participants.

Data collection instruments and analysis

Data collection was guided by study instruments designed by qualitative experts in English, which were translated to Kinyarwanda (see supplementary files). Interviewers used an audio recorder to record the semi-structured interviews and focus group discussions. Recordings were transcribed, and translated into English before being uploaded to QSR International's NVivo 12 qualitative data analysis software for analysis (14). Thematic analysis of data was performed, as well as inductive and deductive coding. Themes were developed iteratively

For peer review only

with reference to the Four Delay framework and according to whether they represent the delay from seeking, reaching, receiving, or remaining in care (9). We defined the first delay (delay in seeking care) as delays from the time the injury occurred to the time a decision was made to seek medical care. The second delay (delay in reaching care) referred to delays from the time a decision was made to seek medical care to the time when an appropriate medical facility capable of managing the particular injury (prior to registration) was accessed. The third delay (delay in receiving care) was defined as delays beginning from the point of entering a capable facility to the point of receiving specific management for the injury. This could include damage control interventions for the injury initiated upon admission to a hospital intended to manage an injured patient's condition or injury. This specific management can be operative surgical care when there is the indication for surgery, but is often non-operative, like the application of casts, administration of medications, or admission for observation. The fourth delay (delays in remaining in care) was defined as delays from the point of having received definitive care to the point of final discharge from follow-up. Remaining in care encompassed the time periods of continued in-hospital care beyond definitive care, discharge, and physical therapy (9). We defined injury as wound or a condition of the body caused by external force or exchange of energy between the body and the environment of such magnitude that is beyond the resilience of the body.

To ensure rigor, all transcripts of interviews and focus group discussions were coded in parallel by a context and local language fluent member (PN) and qualitative data experts (AI and MLO). The analysis team met on a regular basis to discuss the coding process. Any conflicts that arose during the independent coding process were resolved by group consensus. The qualitative analysis procedure was the same for interviews and focus groups. Following coding and the identification of initial categories, data from interviews and focus groups were

For peer review only

combined. The final list of themes was reviewed and agreed upon by the entire investigator team. Barriers were included based on single mention.

RESULTS

A total of 51 participants, 28 from the rural Burera District and 23 from the urban Kigali city, participated in the interviews and focus group discussions. Of all the participants, 13 were female and 38 were male, ranging from 21 to 68 years of age. Thirty-six (71%) of the participants were patients (service users), while the rest were caregivers and community leaders. For the individual in-depth interviews, 10 patients (50%) were from urban Kigali, and 10 (50%) from rural Burera. They presented with a wide range of injuries including fractured long bones (9, 45%), other fractures (3, 15%), head injury (3, 15%), polytrauma (3, 15%), abdominal trauma (1, 5%), and lacerations (1, 5%). The major mechanism of injury was involvement in road traffic collisions in the urban setting (9, 90%), and less so in the rural settings (5, 50%). There were more falls from heights in the hilly rural settings (4, 40%), than in the urban areas. Only 1 rural report of assault was captured.

Sixty barriers were identified across all four delays. The most mentioned barriers were the insufficient number of competent trauma care providers, the rigid referral process, and facility-related delays in delivery of care. Table 1 shows the barriers to injury care identified by participants across all delays. Figure 1 is a word cloud that pictorially represents the barriers to injury care based on mentions in the individual in-depth interviews.

Table 1: Barriers to injury care identified by participants across all delays

**First delays: delays in seeking care**
• *Community health workforce related delays*: Perceived sufficiency of respondent's

For peer review only

interventions.

- *Community leadership related delays*: Community conflict management process.
- *Health financing related delays*: Pre-injury financial challenges and limited comprehensiveness of the insurance system.
- *Health services delivery related delays*: Perceived inaccessibility of the “right” hospital or acute care facility, lack of awareness of healthcare and injury care systems, lack of confidence in healthcare system, and the reference for alternative healthcare.
- *Knowledge on health information related delays*: Underestimation of the severity of the injury, religious beliefs, respondent rumors about seeking injury care, shared decision-making (contribution of community health workers and village leaders).

Second delays: delays in reaching care

- *Health care services access related delays*: Limited geographical coverage of ambulance services, cost of transport to hospital and between hospitals, unavailability of transportation, limited access to ambulances, ignorance of ambulance call process and emergency numbers, high urban road traffic density, and time of the day of injury.
- *Infrastructure related delays*: Poor road infrastructure.
- *Health financing related delays*: Fear of spending on private transport cost and absence of personal health insurance.
- *Community leadership related delays*: Unwillingness of passers-by to assist, fear of legal complications for intervening private car owners, community organization required for reaching facilities, lack of social assistance and support, and accident investigation process by the police.
- *Community health workforce related delays*: Delays in arrival of emergency respondents, occurrence of multiple road traffic accidents, poorly coordinated inter-facility transfers.

Third delays: delays in receiving care

- *Health financing related delays*: Lack of financial means, absence of health insurance, cumbersome facility invoicing and cost recovery system, unavailability of some community health insurance staff during work hours, unaffordable costs of materials and medications purchased in non-government funded facilities, and the inability to use community health insurance in private pharmacies.
- *Health services delivery related delays*: High patient load and limited capacity of

For peer review only

facilities, inadequate facility infrastructure, inadequate follow up of privately referred patients to the point of receiving care by referring physicians, and the reduced staff after daylight working hours.<br>• *Health workforce related delays*: Insufficient skilled medical and specialist staff, unavailability of certain specialized services and the high cost of these services outside the country, poorly organized inter-facility referral system, and traditional healers and their interface with the health system.<br>• *Health information related delays*: Difficulty in the use of electronic medical systems by healthcare staff.<br>• *Medical products related delays*: Unavailable equipment within facilities, and unavailable medications or other treatment within facilities.
**Fourth delays: delays in remaining care**
• *Health services delivery related delays*: Poor counseling of patient in need for rehabilitation by healthcare workers, previous unsatisfactory experience of health facility care, perceived early discharge in an unsatisfactory health condition, complications of alternative home-based care which may limit patients' movement, limited opening hours of some facilities, cumbersome referral system for gaining appointments and multiple transfers required for community health insurance users, lack of triage services at receptions; Slow service delivery at health centers, and difficult follow-up process for discharged patients.<br>• *Health workforce related delays*: Traditional healers counseling patients against returning for follow up, multi-tasking by healthcare workers and lack of staff supervision and many responsibilities on one staff in health centers, and insufficiency of staff and decrease in confidence at HC.<br>• *Health information related delays*: Use of paper-based health records.<br>• *Transport related delays*: Lack of transportation for follow-up, and difficult terrain between home and location for rehabilitation or physiotherapy.

Figure 1: Word cloud of barriers to injury care in Rwanda

Fig1

For peer review only

Delays in seeking care

A typical rural road accident scene would be crowded with bystanders observing the injured and discussing their presumed prognosis. Others at the site of injury would attempt to provide first aid, call the ambulance service and the police, reach out to any available family members using the injured individual's cell phone, manage traffic movements at the crash site, and obtain any information required by the police for post-accident legal procedures. The decision to move victims to a nearby health facility would usually be made after some (usually intense) discussion among bystanders. In the rural setting, the most proximate facilities are typically health centers and health posts and participants mentioned that only a small proportion of the injured would delay seeking care and return home for self-management or seek the services of a traditional healer.

At the scene of an accident, it was reported that in most cases, the initial decision to seek care was deferred by victims or bystanders on account of distance and time to health facilities from the accident location, lack of financial means including considerations of whether or not injured individuals had community health insurance and reticence about going through the process of activating community health insurance payments.

"*(...) when the patient doesn't have health insurance and doesn't have money, they fear going to health facilities as it is complicated. In Kigali city, it is even more complicated as you may go to the hospital and they ask you to pay around 50 to 60 thousand, and it is complicated. Then people may miss going to health facilities due to that." (FGDPatUrban01)*

Furthermore, a poor understanding of the trauma victim's health condition and the need for care, coupled with an inaccurate perception of the severity of injury by the individual and by-standers, often delayed the decision to seek care. Occasionally, community health workers

For peer review only

and village leaders would contribute to this delay if they felt the injury was not severe enough to necessitate care.

"… (..) *a case of a mother who had a child who was kicked against the wall by the sheep, and they said it was not serious, saying that it was normal for the children to fall and have minor pain, until the child continued to swell to the level that they decided to take him to the hospital very late." (FGDPatRural01)*

Socio-cultural and religious beliefs opposed to orthodox healthcare and prior positive experiences with traditional medicine use also contributed to this delay.

"What I also observed is that an individual may have an injury causing bleeding, and instead of taking him to the hospital, they choose to apply herbal medications and later go to the hospital with complications." (FGDPatRural01)."People's belief systems and confidence in traditional medicine contribute a lot to the decisions they take on what to do after having an accident." (FGDComUrban01)

In rural Rwanda, it is the community and family that often decide whether or not an injured member should receive care at a healthcare facility. This shared decision-making process may further delay seeking care. In cases of violent communal conflict, the community conflict management process also delays the decision to seek care, as settling the conflict is prioritized over taking decisions to take victims to a care facility.

"*In the community (…) the members of the wider community first come to see your husband or wife and the family then collectively decide to take you to the health facility.*" *(FGDComRural01)*

Delays in reaching care

For peer review only

The injured individuals would usually be transported in traditional stretchers and on motorbikes, depending on their condition. In contrast, injured in urban Kigali would be typically carried by emergency services ambulances to district and referral hospitals within the city.

In the rural setting, they reported utilizing different modes of transportation from accident scenes to nearby health facilities. This ranged from walking on foot, riding commercial motorcycles, using traditional stretchers (see Figure 2), ambulances and renting private cars. The most common means were commercial motorcycles, if the condition of the injured permitted, and traditional stretchers carried by four members of the community were reserved for severely injured individuals in the rural setting.

Figure 2: A Rwandan traditional stretcher.

Fig2

Photo by Barnabas Alayande.

Only a few of the injured people reported reaching the health facilities by ambulances. The perception was that ambulances were scarce and were only used to transport pregnant women and children from home to hospital. Furthermore, in the rural areas, most people believed that the ambulance were only used for inter-facility transfer and not for initial transport to the hospital.

"I know that you have to first take your patient to the primary health facility, and if they decide to refer him, it is then the time they can call an ambulance." (FGDPatRural01)

For peer review only

This was in contrast to urban participants who reported the use of ambulances as the primary means to reach health facility. Alternatives in Kigali were only used when ambulances were not required, available or accessible.

"... I remember the last time an accident occurred, and there were so many accidents (....) Then there was the surprise of how they instructed the ambulance to deviate its initial direction to pass there and bring one medical personnel at the accident scene to return later after taking that first patient to the hospital." (FGDComUrban01)

Furthermore, the cost of private transport and the unwillingness of private drivers to assist in transporting the injured contributed to the delay in reaching the care. Private car owners, in particular, if they experienced complicated reception processes at health facilities in the past, were not willing to help transport the patients

(...) apart from having a human heart, people are hesitant to help in that situation because bringing the accident victim to the medical facility suggests that you are willing to spend your time there." (FGDComUrban01)

Additionally, high traffic density during rush hour in Kigali, the occurrence of simultaneous road traffic accidents, and COVID-19 curfew-related delays were cited.

"The most challenge is traffic jam, the ambulance may come on time but the road is busy with traffic jam." (FGDPatUrban01) and *"during COVID-19, when there were curfew hours and you wanted to reach the hospital, the motorcycle rider would ask you for a lot of money, saying that he would face the traffic police and that he would need to use shortcut roads which are not safe. If you were lucky, he would take you to the hospital (...) [but] you had to pay a lot of money, almost three times the normal cost since you had no alternative." (FGDPatRural01)*

For peer review only

The absence of personal insurance also led to delays in reaching care.

"… [a] patient who has personal financial challenges (…) advised me to first solve issues of community health insurance before going to the hospital to get care." (PatRural002)

The inter-facility referral and transfer process also contributed to delays in receiving care, especially when it came to asking trauma victims to reach a higher-level facility unassisted and not in the ambulance.

"From the health center, they gave us a referral to [name of district hospital] without facility transport. Here, they also provided us with basic healthcare and a transfer to Ruhengeri hospital [referral hospital], but we had to do it ourselves. We needed four people to lift her [the injured patient] as she is old; it required me to take four motorcycles, one for her, one for me, and others for the ladies to care for her on each trip"(FGDPatRural01)

Delays in receiving care

Most of the participants in rural Burera first received care at the health post or the health center level, while those in Kigali City who used mostly emergency ambulance services to reach care, commonly accessed higher levels of care through district hospitals and the University Teaching Hospital of Kigali (CHUK). However, across the board, the required medication and diagnostic equipment was not always available at these health facilities, and the prohibitive cost of trauma care materials and medications purchased outside of the government facilities delayed receiving care. Facility overload also emerged as a barrier, as hospitals had limited capacity to attend to several patients at once.

"(…) patients with multiple fractures come in and are laid on the ground. The hospital has a serious problem of shortage of beds and they do not have space to put all emergencies they

For peer review only

receive." (FGDPatUrban01). "(...) the infrastructure we have doesn't meet the need as you may find, even the doctors are few. Patients they have to operate on don't have enough space for post-operative care, and this may limit services". (FGDComUrban01)

Some participants experienced delays in receiving care within the facility because of an insufficient number of skilled trauma care providers or specialists. One of the patients interviewed talked about experiencing a delay in receiving care because healthcare providers did not recognize the severity of their injuries.

"(...) they have low skills (...) like in case of fracture. You see, a general physician may not have enough skills on it, and you see them discussing it, and you hear them. They delayed doing this and you had to do this; you know, medical staff, the one who came first [to see me] was clearly working in slow-motion for unjustified reasons." (PatRural004)

Several participants mentioned the organization and flow of patients in the health facility process as being responsible for delays in receiving care. Specific barriers included gaps in the electronic medical records, a lengthy invoicing and record recovery system; difficulties in effecting payments; inadequate communication by healthcare providers coupled with the patient's lack of familiarity with the health facility and long or short duration of treatment.

"*The patient should come and not be required to wait on those benches. They should go immediately to the cashier to pay and see their doctor. It would be better than sitting on those benches, getting a number before going on to pay. Furthermore, some people are assisted by security staff during the payment procedure. Even if you come first, you run the risk of going home unattended by the doctor, as they assist many people." (FGDPatUrban01)*

Another patient said *"I was discharged on Friday, but the process could not be completed that day, and some services were unavailable on Saturday and Sunday, so I had to wait until*

For peer review only

Monday. I believe they should create a shortcut so that the person who discharge you can use their technology to show the credit you have and pay directly." (PatUrban002)

Additionally, *"People have a problem when they discharge them and no longer consider their situation.... you observe for a few days, they discharge you. They released me while both of my legs were stiff from the prolonged suspension." (FGDPatUrban01)*

Furthermore, issues surrounding insurance were cited by participants as causing delays at the facility. Patients with community health insurance reported that insurance staff were not always available to attend to patients during work hours, especially at the primary level of care.

"(...) I saw it in two facilities. You meet him [the insurance staff], and a short while later, when you return to see him, he is not at his workstation, and you have to wait while you are severely suffering. It is mandatory to see him as you cannot be attended to if the insurance worker does not help you." (PatUrban001)

Within public facilities, uninsured patients and those without financial means to purchase prescribed medication faced delays resulting from a relatively high direct cost of care and a long payment process that involved seeking hospital social and administrative approval to secure prescribed medication on credit. Outside government facilities, such as community pharmacies, health insurance was not accepted and the high cost of purchasing medications out of packet was a barrier to receiving care.

"If you have an accident and they have to put in the left leg prosthesis and they don't have it here, they send you to buy it for around 50,000 to 100,000 Rwandan Francs, while if you find it here, you pay only 10,000 Rwandan Francs [with community health insurance]." (FGDPatUrban01). "*Whenever they send you outside [of the health facility to private*

For peer review only - http://bmjopen.bmj.com/site/about/guidelines.xhtml

pharmacies], regardless of your insurance status, the amount of money billed for medication will be too much for you (FGDPatUrban01)"

Delays to remaining in care

Most participants felt injured patients had been discharged before full recovery due to insufficient beds and the need to admit other trauma patients. This led to difficulties in returning for follow-up as the patient's condition was not optimal for the strain of the travel and the visit.

"*Often, the patient is discharged before achieving total recovery (...) the doctor decides to continue following up with the patient while at home. In that situation, the patient may not be satisfied." (FGDComUrban01)*

"(...) the long travel on foot made it swollen, and I reached there with the arm all swollen. I had to be there early, an hour and a half before any physical examination, so that the swelling would go down and the doctor would not be confused. I don't want him to think that I have another problem while the reason for the swelling was actually from traveling for the appointment." (PatUrban010)

While agreeing that they had been given follow-up and rehabilitation appointments, the distance from home to rehabilitation facilities, difficulties in arranging transport, and the cost of transport were common barriers to returning. Some injured patients visited traditional healers and were subsequently dissuaded from attending the follow-up appointments in the health facility or hospital.

Most of the interviewed participants were community health insurance users. They identified delays caused by the need for patients to present a new transfer from lower levels of care

For peer review only

before being attended to for follow-up at higher levels of care from which they were discharged. Presenting for rehabilitation or follow-up at a tertiary facility necessitates returning to the health center and district hospital to ensure insurance claim payment by obtaining a new referral or transfer for each visit that exceeds 30 days following discharge.

"When I was discharged, I came back [for my follow-up appointment] and stayed there till 3:00 pm. When I finally reached those cashiers, they asked me for the transfer, and I said I had an appointment. They insisted I needed the transfers. I asked them why because I was previously admitted, and they told me to go back to the health center and then to the district hospital to get the transfers and to reschedule my appointment for later. I (...) finally went back home, then to the health center and from there to the district hospital before finally coming back again to ask for a new appointment." (FGDPatUrban01)

DISCUSSION

This study is the first qualitative study, of which we are aware, to identify barriers to injury care from the point of injury to rehabilitation in both rural and urban settings in Rwanda, utilizing the four delays framework. A recent systematic review found that very few studies in lower- and middle-income countries have comprehensively assessed barriers to access to quality trauma care (8) from being injured to being discharged from care. What is more, we focused on urban and rural settings in recognition of possible disparities that may exist in accessing injury care in Rwanda. Multiple barriers to injury care in were identified across the four delays, in both rural and urban settings. The barriers most mentioned by patients and community leaders were "insufficient number of competent trauma care providers," "rigid referral process," and "facility-related delays in delivery of care." These findings echo the results of a multidisciplinary stakeholder consultation aimed at mapping injury care access in Rwanda, which found that "training and retention of specialist staff," "geographical coverage

For peer review only

of referral trauma centers," and "lack of protocol for a bypass to referral centers" among the top 4 barriers identified by the participants (9). The similarity of findings from these two studies, despite the differing perspectives of contributors, suggests that interventions aimed at ensuring access to safe, timely, affordable and quality injury care should take into consideration these specific barriers.

Regarding transport for injured patients to facilities that can manage their injuries, a limited number of ambulances available to convey injured patients from the point of injury to the appropriate trauma care facility was identified as a cause of delay. A systematic review of literature in LMICs found that often ambulances were not available, but even when ambulances are available, many emergency and trauma patients are forced to use alternative means of transport (15). Rwanda is relatively unusual in sub-Saharan Africa in having a country-wide ambulance service. However, discussions at the aforementioned workshop and in the 2019 Rwanda trauma symposium, highlighted that efficiency could be improved with better location of the patient and communication between the ambulances and facilities (9)(16). Studies are ongoing to address these issues(17)(18). Although our participants in Kigali stated that injured people often used ambulances, it was clear that their availability was much lower in rural areas. Rwanda's unique hilly topography must be considered when implementing low-cost and innovative ambulance services for rural populations. Another perspective, however, may be that participants did not know how to access the ambulance service. The widespread perception in this study was that in rural areas, ambulance use was restricted to only transporting pregnant women or sick children from home to hospital. Rwanda's Service d'Aide Medicale d'Urgence (SAMU) was founded in 2008 and now has up to 225 ambulances (five per district). This results in an ambulance to population ratio of 1:48,000 and exceeds the World Health Organization (WHO) recommendation of 1:50,000 (15). The rural-urban disparity in the perception of the availability of ambulances for injury

For peer review only

care should be addressed. Community education, alongside improved coordination and logistics of ambulance distribution for injury care, should to be optimized (19) (20)

In Sub-Saharan Africa, surgical and anesthetist densities are generally low. By 2015, Rwanda had only 0.79 per 100,000 skilled health workers, a significantly lower ratio than in High-Income Countries HICs (21). Trauma care providers are perceived to be in short supply and inequitably distributed in favor of urban areas. The participants in this study identified "insufficient number of qualified trauma care providers" as a major barrier to receiving injury care. Hutch et al. found that only 20 surgeons were trained by the College of Surgeons for East Central and Southern Africa in the 13 years leading to 2013(22). Despite this low number of surgeons, Rwanda has a high level of surgeon retention (90%) following training (22). However, this retention is particularly skewed towards urban areas, and an attrition rate of up to 80% for doctors in the rural setting has been identified (23). Rwandan anesthesiologists and surgeons with specialized training work almost entirely in urban referral hospitals, leaving surgical care at rural district hospitals largely to general practice physicians and nurses (24). The presence of trained anesthetists and the availability of anesthesia are essential to operative injury care. A 2017 study found that over a third of anesthetists trained in Rwanda had emigrated for a variety of reasons, ranging from a desire for better remuneration to a lack of equipment and medication for safe anesthesia, occupational isolation in rural settings, and demoralization (25). Only about 75 physiotherapists serve Rwanda's entire population of over 13 million people (26). This issue of insufficient trauma care providers is in keeping with findings from other LMICs. In a systematic review of barriers to out-of-hospital emergency trauma care, over 60% of them identified a lack of skilled personnel as a key barrier (15); lack of available, trained, and motivated clinical staff was the second highest barrier to access to quality trauma care identified in an international Delphi exercise(20). The WHO recognizes the role of various

For peer review only

levels of healthcare delivery in trauma management. Non-physician providers and non-specialist providers (general practitioners) in low-income countries are responsible for a large portion of trauma care (27). Therefore, human resource development for injury care and appropriate staffing should be prioritized at all levels of healthcare delivery alongside the provision of required infrastructure, equipment, and supplies to facilitate the better delivery of trauma care (27). Models such as Rwanda's Human Resource for Health program should be improved upon and strengthened to optimize trauma care specialist training (28). Training and retention of surgeons and anesthetists, particularly in rural areas, should be encouraged and incentivized (29).

Rwanda has one of the most robust health insurance population coverages in sub-Saharan Africa, with community-based health insurance held by over 91% of individuals(30). This facilitates much needed access to care for those who have insurance. However, it is not yet comprehensive enough to benefit all injured patients (9)(31)(32). Participants in both interviews and focus group discussions mentioned barriers related to community health insurance. Multiple insurance-related referral processes, long payment procedures within public health facilities for insurance holders, and non-acceptance in private pharmacies contributed to the delays. Additionally, procedures to prove possession of insurance meant that many patients in our study experienced further delays. The dissatisfaction with the procedures for checking insurance has been cited as one of the major barriers to community-based health insurance renewal (33), despite the fact that 97% of those in the scheme say they benefit from it (31). For the community health insurance to have a strong positive impact on access to healthcare, those limitations have to be addressed (32), and more efforts should be engaged toward the development of prehospital and inter-facility transportation arrangements, strengthening district hospitals, and better supporting referral institutions (34).

For peer review only

Many participants in this study reported being discharged before they perceived that they were fully recovered. They believed that the early discharge was due to insufficient bed space. Early mobilization and discharge maybe advantageous for trauma patients to reduce complications of deep vein thrombosis, joint stiffness, and depression, among others (35). However, this early discharge was not complemented by the availability of decentralized rehabilitation services. To be able to return to the treating tertiary facility for physiotherapy or other rehabilitation services for appointments beyond the 30 days allowed by community health insurance, the patients in our study were asked to go through the entire referral process again, without which their insurance payments would not be guaranteed. This involved returning to the initial referral health center, then to the next district facility that had referred the patient, and finally to the tertiary facility with appointments. This time-consuming procedure was viewed as a barrier to continuing in care.

At the University Teaching Hospital of Kigali, up to 84% of orthopedic trauma patients (most of whom required post-discharge rehabilitation) have been found to come from facilities in other provinces rather than Kigali. This pattern results in a heightened need for inter-facility transfer (34). Our study suggests that a simplified pathway to follow-up care for injury patients may help reduce losses to follow-up. In addition, options for home-based primary care and other innovative methods may need to be considered and tested (36), based on the needs and preferences of the communities. These reflections align with other studies. For example, using an online questionnaire distributed through a variety of international and regional organizations from the various WHO regions, Darci et al. found that 31.8% of respondents viewed providing rehabilitation services within specialized hospitals and units completely unacceptable, relative to general hospitals or non-specialized units. Up to 79.3% of the same respondents preferred community-based rehabilitation over hospital-based rehabilitation (37).

For peer review only

This study is not without its limitations. Whilst Kigali is the major conurbation in Rwanda, it is not the only one and our findings from Kigali may not be representative of other urban settings. Likewise, the rural interviews were conducted only in Burera, and certain nuances of injury care access within rural Rwanda may have been missed because of the wide variation in rural settlements while we recognize that barriers may differ slightly by district setting. Additionally, most (75%) of the patients were male, whilst this may mean that we missed barriers mostly experienced by females, it should be noted that the majority of injured patients in Rwanda are male. In addition, this study did not ensure a range of injury by anatomical/pathological types, severity, mechanism across participants, which may have helped to elicit specific barriers. However, the urban and rural spread of participants was used as a proxy for inclusion of injuries by socioeconomic status. It must also be noted that the 3- and 4-delay frameworks have been critiqued as being too simplistic (38). The model has been critiqued for being too unidimensional (39) and sequential (40) in contrast to more sophisticated delay models like the access to healthcare conceptual model that provides a richer and more detailed set of factors and interactions (41)(42). The 4-delay framework may miss the complex reality of real-life scenarios and is not directly focused on identifying potential solutions.

CONCLUSION

In contrast to previous studies that focused primarily on the delay to receiving care in health facilities, this qualitative study provides insights into barriers to seeking, reaching, receiving and remaining in care from the injured patients', caregivers' and community leaders' perspectives. In both rural and urban settings, hindrances along the spectrum of care, from injury to rehabilitation, and at various levels of the health system, have been identified. These barriers should be considered and guide injury interventions and health facility organization.

For peer review only

Human resources for health strengthening specific to injury care, facility re-organization to reduce in-facility waiting times, and revision of the complex follow-up referral system are priority areas for intervention. Future health policy planning should strengthen trauma care by focusing and addressing these barriers.

Ethics approval

The overall study was approved by University of Birmingham Research Ethics Committee, UK (ERN_20-00880). Data collection was approved by the National Health Research Committee (NHRC/2020/PROT/044) for Rwanda. Additional approval was sought from the respective hospitals before visiting the facilities. Community leaders within the facilities' catchment area also gave permissions and participated in the interviews.

Author contributions

CRediT author statement: **Pascal Nzasabimana:** Formal analysis, Investigation, Data Curation, Writing - Original Draft, Visualization, Validation, Formal analysis, Project administration; **Agnieszka Ignatowicz:** Methodology, Formal analysis, Writing - Review & Editing; **Barnabas Alayande:** Formal analysis, Validation, Writing - Original Draft, Writing - Review & Editing, Visualization; **Abdul-Malik Abdul-Latif:** Formal analysis, Writing - Review & Editing; **Maria Lisa Odland:** Formal analysis, Writing - Review & Editing; **Justine I Davies:** Conceptualization, Validation, Resources, Writing - Review & Editing, Supervision, Funding acquisition **Abebe Bekele:** Writing - Review & Editing, Supervision **Jean Claude Byiringiro:** Conceptualization, Resources, Writing - Review & Editing, Supervision, Funding acquisition

Data sharing statement

For peer review only

The data that support the findings of this study are available upon reasonable request from the corresponding author. The data are not publicly available due to restrictions as they contain information that could compromise the privacy of research participants.

Competing interest: None declared

Word count: 8059

REFERENCES

1. Anon. Global burden of 369 diseases and injuries in 204 countries and territories, 1990-2019: a systematic analysis for the Global Burden of Disease Study 2019. Lancet (London, England) 2020;396:1204–1222.

2. Obermeyer Z, Abujaber S, Makar M, et al. Emergency care in 59 low- and middle-income countries: A systematic review. Bull World Health Organ World Health Organization 2015;93:577–586.

3. The World Bank. Cause of death, by injury (% of total) - Rwanda. Data 2020. Available at: https://data.worldbank.org/indicator/SH.DTH.INJR.ZS?locations=RW. Accessed June 23, 2022.

4. Kim WC, Byiringiro JC, Ntakiyiruta G, et al. Vital Statistics: Estimating Injury Mortality in Kigali, Rwanda. World J Surg 2016;40:6–13.

5. Edem IJ, Dare AJ, Byass P, et al. External injuries, trauma and avoidable deaths in Agincourt, South Africa: a retrospective observational and qualitative study. BMJ Open 2019;9:e027576.

6. Stewart B, Hollis S, Amato SS, et al. Trauma care and development assistance: Opportunities to reduce the burden of injury and strengthen health systems. Bull World Health Organ 2019;97:371–373.

7. Calvello EJ, Skog AP, Tenner AG, Wallis LA. Applying the lessons of maternal mortality

For peer review only

reduction to global emergency health. Bull World Health Organ 2015;93:417–423.

8. Whitaker J, O'Donohoe N, Denning M, et al. Assessing trauma care systems in low-income and middle-income countries: A systematic review and evidence synthesis mapping the Three Delays framework to injury health system assessments. BMJ Glob Heal 2021;6:1–15.

9. Odland Maria Lisa, Dmitri, Achieng, Nepogodiev John C, et al. Identifying , Prioritizing and Visually Mapping Barriers to Injury Care in Rwanda : A Multi-disciplinary Stakeholder Exercise. World J Surg 2020;44:2903–2918.

10. Odland ML, Abdul-Latif A-M, Ignatowicz A, et al. Equitable access to quality trauma systems in low-income and middle-income countries: assessing gaps and developing priorities in Ghana, Rwanda and South Africa. BMJ Glob Heal 2022;7:e008256.

11. National Institute of Statistics of Rwanda (NISR). 2012 Population and Housing Census (PHC) Brochure. 2012 Population and Housing Census. Kigali; 2013:264.

12. Laterite. The economic geography of Rwandan cities. Kigali; 2019.

13. Rwanda Ministry of Health. Fourth Health Sector Strategic Plan. Ministry of Health. 2018.

14. Lyn Richards. Handling Qualitative Data: A Practical Guide. Sage Publ. xii:207. Available at: https://www.qualitative-research.net/index.php/fqs/article/view/107/223. Accessed June 16, 2022.

15. Kironji AG, Hodkinson P, De Ramirez SS, et al. Identifying barriers for out of hospital emergency care in low and low-middle income countries: A systematic review. BMC Health Serv Res 2018;18:1–20.

16. Rosenberg A, Ntirenganya F, Bagahirwa I, et al. First Rwanda National Trauma Symposium 2019: Challenges and priorities. J Glob Health 2020;10:1–8.

17. Ashley Rosenberga, Rob Rickardb, Fraterne Z. Uwinshutic, Gabin Mbanjumucyod,

For peer review only

Menelas Nkeshimanad, JeanMarie Uwitonzec, Ignace Kabagemac, Theophile Dushimec SJ. Collaboration for preliminary design of a mobile health solution for ambulance dispatch in Rwanda. 2020.

18. Jayaraman S, Ntirenganya F, Nkeshimana M, et al. Building trauma and ems systems capacity in rwanda: Lessons and recommendations. Ann Glob Heal 2021;87:1–13.

19. Kannan VC, Tenner A, Sawe HR, et al. Emergency care systems in Africa: A focus on quality. African J Emerg Med 2020;10:S65–S72.

20. Whitaker J, Nepogodiev D, Leather A, Davies J. Assessing barriers to quality trauma care in low and middle-income countries: A Delphi study. Injury Netherlands 2020;51:278–285.

21. Ministry of Health. Health Labor Market Analysis Report. 2019.

22. Hutch A, Bekele A, O'Flynn E, et al. The Brain Drain Myth: Retention of Specialist Surgical Graduates in East, Central and Southern Africa, 1974–2013. World J Surg 2017;41:3046–3053.

23. Odhiambo J, Rwabukwisi FC, Rusangwa C, et al. Health worker attrition at a rural district hospital in Rwanda: A need for improved placement and retention strategies. Pan Afr Med J 2017;27:1–7.

24. Notrica MR, Evans FM, Knowlton LM, Kelly McQueen KA. Rwandan surgical and anesthesia infrastructure: A survey of district hospitals. World J Surg 2011;35:1770–1780.

25. Skelton T, Irakoze A, Bould MD, et al. Retention and Migration of Rwandan Anesthesiologists: A Qualitative Study. Anesth Analg 2020;131:605–612.

26. World Physiotherapy. Rwanda Physical Therapy Organisation. Rwanda profile 2020. Available at: https://world.physio/membership/rwanda. Accessed February 24, 2022.

27. Dries DJ. Guidelines for Essential Trauma Care. Shock 2005;23:97–98.

28. Binagwaho A, Kyamanywa P, Farmer PE, et al. The Human Resources for Health Program in Rwanda — A New Partnership. N Engl J Med 2013;369:2054–2059.

For peer review only

29. Van Essen C, Steffes BC, Thelander K, et al. Increasing and Retaining African Surgeons Working in Rural Hospitals: An Analysis of PAACS Surgeons with Twenty-Year Program Follow-Up. World J Surg 2019;43:75–86.

30. Kalisa, Musange, David S& K. The Development of Community-Based Health Insurance in Rwanda: Experiences and Lessons. Univ Rwanda, msh, Rockefeller Found 2016;1.

31. David Collins US. The Impact of Community-Based Health Insurance on Access to Care and Equity in Rwanda BACKGROUND. Manag Sci Heal MSH 2016:1–8.

32. Saksena P, Antunes AF, Xu K, et al. Mutual health insurance in Rwanda: Evidence on access to care and financial risk protection. Health Policy (New York) 2011;99:203–209.

33. Hussien M, Azage M. Barriers and facilitators of community-based health insurance policy renewal in low-and middle-income countries: A systematic review. Clin Outcomes Res 2021;13:359–375.

34. Ntakiyiruta G, Wong EG, Rousseau MC, et al. Trauma care and referral patterns in Rwanda: Implications for trauma system development. Can J Surg 2016;59:35–41.

35. Kang Y, Liu J, Chen H, et al. Enhanced recovery after surgery (ERAS) in elective intertrochanteric fracture patients result in reduced length of hospital stay (LOS) without compromising functional outcome. J Orthop Surg Res 2019;14:1–7.

36. Rezaei M, Sharifi A, Vaccaro A, Rahimi-Movaghar V. Home-based rehabilitation programs: Promising field to maximize function of patients with traumatic spinal cord injury. Asian J Neurosurg 2019;14:634.

37. Darzi AJ, Officer A, Abualghaib O, Akl EA. Stakeholders' perceptions of rehabilitation services for individuals living with disability: A survey study. Health Qual Life Outcomes 2016;14:1–9.

38. Bohren MA, Hunter EC, Munthe-Kaas HM, et al. Facilitators and barriers to facility-based delivery in low- and middle-income countries: A qualitative evidence synthesis.

For peer review only

Reprod Health 2014;11:1–17.

39. Sorensen BL, Nielsen BB, Rasch V, Elsass P. User and provider perspectives on emergency obstetric care in a Tanzanian rural setting: a qualitative analysis of the three delays model in a field study. Afr J Reprod Health 2011;15:117–130.

40. D'Ambruoso L, Byass P, Qomariyah SN, Ouédraogo M. A lost cause? Extending verbal autopsy to investigate biomedical and socio-cultural causes of maternal death in Burkina Faso and Indonesia. Soc Sci Med Elsevier Ltd 2010;71:1728–1738.

41. Khan AA, Bhardwaj SM. Access to Health Care: A Conceptual Framework and its Relevance to Health Care Planning. Eval Health Prof SAGE Publications Inc 1994;17:60–76.

42. Combs Thorsen V, Sundby J, Malata A. Piecing Together the Maternal Death Puzzle through Narratives: The Three Delays Model Revisited. PLoS One 2012;7.

For peer review only

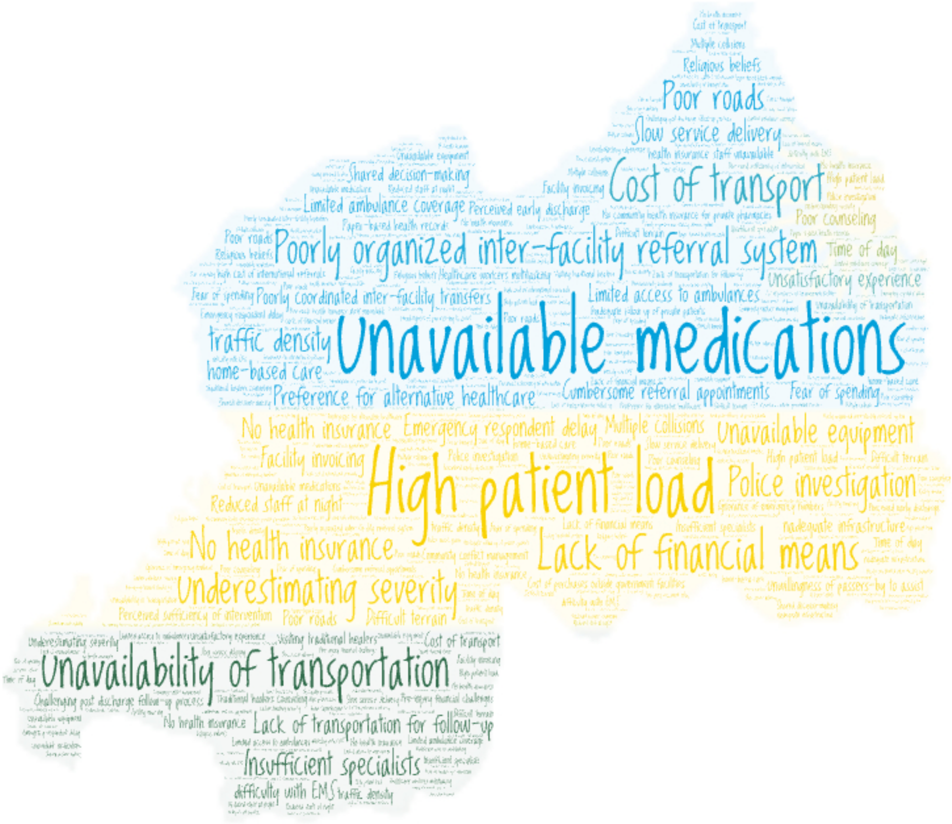

Figure 1: Word cloud of barriers to injury care in Rwanda

268x231mm (72 x 72 DPI)

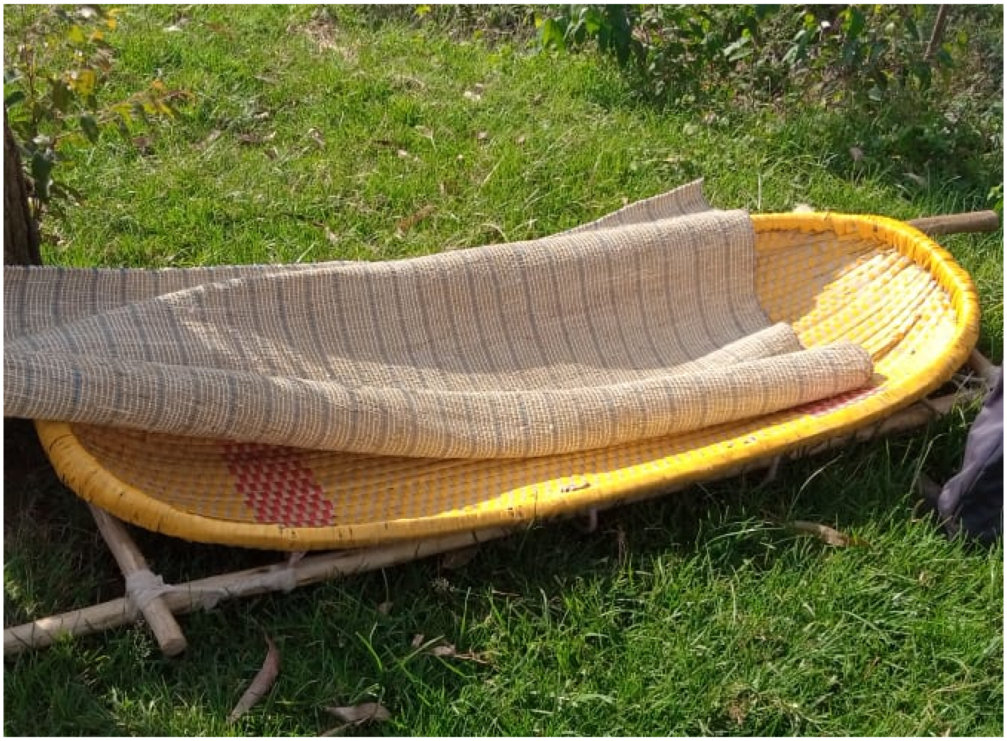

Figure 2: A Rwandan traditional stretcher.

17119x12547mm (1 x 1 DPI)

Supplementary Information

Discussion Guides

1. Service Users Focus Group Discussion Guide

First, I would like to thank you for attending this discussion.

The recordings will be anonymous, and we don't want you to say your name at the start, but we have given each of you a pseudonym (made up name), and will go around the table in turn asking you to say your pseudonym, what age you are, the area in which you live, This will help us to identify individual voices when we come to write up the interviews.

This group discussion is to understand what would happen in <u>your community</u> if someone suffered an injury.

We would like to think particularly about four different aspects of what would happen, these aspects are <u>seeking care</u> (the time from an injury happens to taking action to get care), <u>reaching care</u> (the time until a facility or hospital that can treat the injury is reached), <u>receiving quality care</u> (getting good quality treatment for the injury at a facility or hospital), and <u>remaining in care</u> (for example, attending follow up at out-patients or going to rehabilitation).

Seeking care

Firstly, we wish to know what do people do straight after injuries occur?
Does the severity of the injury (how bad it is) determine what people do?

For people who need to go and get treatment, what happens after injuries?
What factors do you think make people more likely to seek health care?
What factors do you think make people less likely to seek health care? <u>Prompts, if necessary</u> For example:

- Knowledge of availability of healthcare?
- Trust in healthcare?

For peer review only

- Fear of costs?
- Availability of a phone?

If people don't seek healthcare what do they do instead?

Reaching Care

Now we wish to know what a person does after an injury has occurred and it has been decided to seek health care.
Does what a person does depend on the severity of the injury?

What factors would make it more likely to reach a place of care quickly?
What factors make it less likely to reach a place of care quickly? Prompts, if necessary For example:

- Ambulance availability?
- Road conditions?
- Personal safety?
- Costs?

Receiving care

How about the care received after the injured person has made it to a place of care? Would you consider that this will be good quality care? Prompts, if necessary For example:

- Will the injured person get the correct treatment?
- Will they be treated with respect?
- Will they have to wait for a long time?

What factors do you think make it difficult for injured people to receive good quality care? What factors make it more likely they will receive good quality care? Prompts, if necessary For example:

- Qualifications of staff at the facility?
- Availability of equipment at the facility?

For peer review only

- Personal contacts with staff at the facility?
- Costs of care?

Remaining in care

This is the final stage in the care-seeking process and we would like you to tell us about what happens for people who are discharged from hospital or, when their injury has been mostly treated, but, they have been asked to come back for more care, like out-patient appointments or physiotherapy (rehabilitation)

Do most people who have been asked to return to (or remain in) care actually do so?
What are the barriers to people remaining in care?
What are the facilitators to people remaining in care?

Prompts, if necessary

For example:

- Distances needed to travel?
- Other family commitments?

Solutions

Finally, what do you think could be done to improve access to quality care for people who are injured in your area?

Does anyone have any questions for us?
Thank you

For peer review only

2. **Service User In-Depth Interview Topic Guide**

First, I would like to thank you for attending this interview.

The recordings will be anonymous, and we don't want you to say your name at the start, but we have given you a pseudonym (made up name) that you will use during this interview.

This interview is to understand what would happen in your community if someone suffered an injury.

I would like to think particularly about four different aspects of what would happen, these aspects are seeking care (the time from an injury happens to taking action to get care), reaching care (the time until a facility or hospital that can treat the injury is reached), receiving quality care (getting good quality treatment for the injury at a facility or hospital), and remaining in care (for example, attending follow up at out-patients or going to rehabilitation).

I wish to know about your experiences and also your thoughts on whether others in your community have similar experiences.

I realize that you have had an injury in the last 6 months, could you tell us about what happened to cause the injury?

Where were you when the injury happened?

How about after your injury happened, what did you do?
Prompts, if needed:

- Did you think that you needed help?
- Was anyone there to help?
- Were you able to telephone for help?

Did you think that you needed to go and get treatment?

For peer review only

(If not, why not?)

Were there any things that stopped you trying to get treatment?

Prompts, if needed

- Knowledge of availability of healthcare?
- Trust in healthcare?
- Fear of costs?
- Availability of a phone?
- No time to go to healthcare?

Where did you first go to for treatment?

Prompts, if needed:

- Is that a clinic or a hospital?
- How far away was it?
- How long did it take to get there?
- If it took a long time to reach the care, why was that?

Was that the only place you went to, or did you need to (or were you referred to) another place to get care?

Note to interviewer: please explore how many places the person needed to go to in order to get definitive care

Were there any things in particular that delayed you getting to a hospital or clinic for care?

Prompts, if needed:

- Ambulance availability?
- Road conditions?
- Personal safety?
- Costs?

For peer review only

How about the care that you got in the facilities that you attended – do you think that the medical treatment was good?

<u>Prompts, if needed:</u>

- Were you seen quickly?
- Did the doctors and nurses seem competent?
- Do you think that they had all that they needed to treat your injury well?

How about how they treated you?

<u>Prompts, if needed:</u>

- Were you treated with respect?
- Did the staff explain things to you?

How about costs of care?

<u>Prompts, if needed:</u>

- Did you have to pay for care?
- Did you have to borrow or sell anything to pay for care?

Were there any things in particular that stopped you getting the right treatment in the hospital?

<u>Prompts if needed:</u>

- Family commitments meant had to shorten stay?
- Costs of care?
- Too far for relatives to travel to visit?

After your injury was treated, were you asked to go back to see healthcare workers about your injury? For example, doctors in a clinical appointment, or physiotherapists for rehabilitation?

If so, how far did you have to travel to get to these appointments?

Did you manage to keep all of the appointments?

For peer review only

Were there any things in particular that you felt limited your ability to keep all these appointments? Prompts, if needed:

- Travel time?
- Costs?
- Other commitments?
- Thought there was no need?

Are you now back to your full fitness, do you think?

Do you think anything could have been done better?

Now that's the end of this discussion, thank-you for telling us about your experiences. Now, we would be grateful if we could ask you some other questions about your time in the hospital or clinic. It will take another ten minutes.

Note to interviewer ask I-PAHC and/or O-PAHC questionnaire depending on whether the participant was admitted to hospital or was seen in a clinic, or both.

Do you have any questions for me?

Thank you

For peer review only

3. Community Leaders Focus Group Discussion Guide.

First, I would like to thank you for attending this discussion.

The recordings will be anonymous, and we don't want you to say your name at the start, but we have given each of you a pseudonym (made up name), and will go around the table in turn asking you to say your pseudonym, what age you are, the area in which you live, and what your job is. This will help us to identify individual voices when we come to write up the interviews.

This group discussion is to understand what would happen in <u>your community</u> if someone suffered an injury.

Please based what you say on what you have observed in your community or experienced yourself.

We would like to think particularly about four different aspects of what would happen, these aspects are <u>seeking care</u> (the time from an injury happens to taking action to get care), <u>reaching care</u> (the time until a facility or hospital that can treat the injury is reached), <u>receiving quality care</u> (getting good quality treatment for the injury at a facility or hospital), and <u>remaining in care</u> (for example, attending follow up at out-patients or going to rehabilitation).

To help you think about this subject, we have provided the following examples:

1) A 19 year old girl who slipped off the roof at home hitting her head on the ground and then became very drowsy.
2) A man who has severe pain after being stabbed in the stomach with a knife after an argument in the street.
3) A farmer who was kicked him in the chest by his cow and has difficulty in breathing.
4) A motorcyclist who was hit by another vehicle has a wound and leg swelling with severe pain.

Seeking care

For peer review only

Firstly, we wish to know what would people do straight after injuries occur?
Does the severity of the injury (how bad it is) determine what people do?

For people who need to go and get treatment, what happens after injuries?
What factors do you think make people more likely to seek health care?
What factors do you think make people less likely to seek health care? Prompts, if necessary For example:

- Knowledge of availability of healthcare?
- Trust in healthcare?
- Fear of costs?
- Availability of a phone?
- Preference for traditional or community healers?

If people don't seek healthcare what do they do instead?

Reaching Care

Now we wish to know what a person does after an injury has occurred and it has been decided to seek health care.
Does what a person does depend on the severity of the injury?

What factors would make it more likely to reach a place of care quickly?
What factors make it less likely to reach a place of care quickly? Prompts, if necessary For example:

- Ambulance availability?
- Road conditions?
- Personal safety?
- Costs?

Receiving care

How about the care received after the injured person has made it to a place of care? Would you consider that this will be good quality care? Prompts, if necessary For example:

For peer review only

- Will the injured person get the correct treatment?
- Will they be treated with respect?
- Will they have to wait for a long time?

What factors do you think make it difficult for injured people to receive good quality care? What factors make it more likely they will receive good quality care? Prompts, if necessary For example:

- Qualifications of staff at the facility?
- Availability of equipment at the facility?
- Personal contacts with staff at the facility?
- Costs of care?

Remaining in care

This is the final stage in the care-seeking process and we would like you to tell us about what happens for people who are discharged from hospital or, when their injury has been mostly treated, but, they have been asked to come back for more care, like out-patient appointments or physiotherapy (rehabilitation)

Do most people who have been asked to return to (or remain in) care actually do so?
What are the barriers to people remaining in care?
What are the facilitators to people remaining in care?

Prompts, if necessary

For example:

- Distances needed to travel?
- Other family commitments?

Solutions

Finally, what do you think could be done to improve access to quality care for people who are injured in your area?

Does anyone have any questions for us?

Thank you

For peer review only

Standards for Reporting Qualitative Research (SRQR)*

http://www.equator-network.org/reporting-guidelines/srqr/

Page/line no(s).

Title and abstract

**Title** - Concise description of the nature and topic of the study Identifying the study as qualitative or indicating the approach (e.g., ethnography, grounded theory) or data collection methods (e.g., interview, focus group) is recommended	Page 1/lines 1-3
**Abstract** - Summary of key elements of the study using the abstract format of the intended publication; typically includes background, purpose, methods, results, and conclusions	Pages 2 and 3

Introduction

**Problem formulation** - Description and significance of the problem/phenomenon studied; review of relevant theory and empirical work; problem statement	Page 4
**Purpose or research question** - Purpose of the study and specific objectives or questions	Page 5/Lines 15-18

Methods

**Qualitative approach and research paradigm** - Qualitative approach (e.g., ethnography, grounded theory, case study, phenomenology, narrative research) and guiding theory if appropriate; identifying the research paradigm (e.g., postpositivist, constructivist/ interpretivist) is also recommended; rationale**	Page 7/Lines 14-16
**Researcher characteristics and reflexivity** - Researchers' characteristics that may influence the research, including personal attributes, qualifications/experience, relationship with participants, assumptions, and/or presuppositions; potential or actual interaction between researchers' characteristics and the research questions, approach, methods, results, and/or transferability	Pages 6 and 7
**Context** - Setting/site and salient contextual factors; rationale**	Page 7
**Sampling strategy** - How and why research participants, documents, or events were selected; criteria for deciding when no further sampling was necessary (e.g., sampling saturation); rationale**	Page 7/Lines 17-24
**Ethical issues pertaining to human subjects** - Documentation of approval by an appropriate ethics review board and participant consent, or explanation for lack thereof; other confidentiality and data security issues	Page 27/Lines 8-12<br>Page 8/Lines 11-14
**Data collection methods** - Types of data collected; details of data collection procedures including (as appropriate) start and stop dates of data collection and analysis, iterative process, triangulation of sources/methods, and modification of procedures in response to evolving study findings; rationale**	Page 8/Lines 1-19

For peer review only

For peer review only - http://bmjopen.bmj.com/site/about/guidelines.xhtml

**Data collection instruments and technologies** - Description of instruments (e.g., interview guides, questionnaires) and devices (e.g., audio recorders) used for data collection; if/how the instrument(s) changed over the course of the study	Page 7/Lines 21-22
**Units of study** - Number and relevant characteristics of participants, documents, or events included in the study; level of participation (could be reported in results)	Page 9/Lines 18-21
**Data processing** - Methods for processing data prior to and during analysis, including transcription, data entry, data management and security, verification of data integrity, data coding, and anonymization/de-identification of excerpts	Page 8/Lines 16-24. Page 9/Lines 1-14
**Data analysis** - Process by which inferences, themes, etc., were identified and developed, including the researchers involved in data analysis; usually references a specific paradigm or approach; rationale**	Page 8/Lines 16-24. Page 9/Lines 1-14
**Techniques to enhance trustworthiness** - Techniques to enhance trustworthiness and credibility of data analysis (e.g., member checking, audit trail, triangulation); rationale**	Page 9/Lines 1-14

Results/findings

**Synthesis and interpretation** - Main findings (e.g., interpretations, inferences, and themes); might include development of a theory or model, or integration with prior research or theory	See table 1: Page 10
**Links to empirical data** - Evidence (e.g., quotes, field notes, text excerpts, photographs) to substantiate analytic findings	Pages 13-21

Discussion

**Integration with prior work, implications, transferability, and contribution(s) to the field** - Short summary of main findings; explanation of how findings and conclusions connect to, support, elaborate on, or challenge conclusions of earlier scholarship; discussion of scope of application/generalizability; identification of unique contribution(s) to scholarship in a discipline or field	Pages 22-26
**Limitations** - Trustworthiness and limitations of findings	Page 26 Lines 13-20

Other

**Conflicts of interest** - Potential sources of influence or perceived influence on study conduct and conclusions; how these were managed	Page 5/Lines 4 and 5
**Funding** - Sources of funding and other support; role of funders in data collection, interpretation, and reporting	Page 5/Lines 1-3

*The authors created the SRQR by searching the literature to identify guidelines, reporting standards, and critical appraisal criteria for qualitative research; reviewing the reference lists of retrieved sources; and contacting experts to gain feedback. The SRQR aims to improve the transparency of all aspects of qualitative research by providing clear standards for reporting qualitative research.

For peer review only - http://bmjopen.bmj.com/site/about/guidelines.xhtml

**The rationale should briefly discuss the justification for choosing that theory, approach, method, or technique rather than other options available, the assumptions and limitations implicit in those choices, and how those choices influence study conclusions and transferability. As appropriate, the rationale for several items might be discussed together.

Reference:

O'Brien BC, Harris IB, Beckman TJ, Reed DA, Cook DA. **Standards for reporting qualitative research: a synthesis of recommendations.** *Academic Medicine*, Vol. 89, No. 9 / Sept 2014 DOI: 10.1097/ACM.0000000000000388

For peer review only

For peer review only - http://bmjopen.bmj.com/site/about/guidelines.xhtml
